# Recipient c-Kit Lineage Cells Repopulate Smooth Muscle Cells of Transplant Arteriosclerosis in Mouse Models

**DOI:** 10.1161/CIRCRESAHA.119.314855

**Published:** 2019-05-13

**Authors:** Zhichao Ni, Jiacheng Deng, Claire M.F. Potter, Witold N. Nowak, Wenduo Gu, Zhongyi Zhang, Ting Chen, Qishan Chen, Yanhua Hu, Bin Zhou, Qingbo Xu, Li Zhang

**Affiliations:** 1 From the School of Cardiovascular Medicine and Sciences, King’s College London, BHF Centre, United Kingdom (Z.N., J.D., C.M.F.P., W.N.N., W.G., Z.Z., Y.H., Q.X.); 2 Department of Cardiology, the First Affiliated Hospital, School of Medicine, Zhejiang University, Hangzhou, China (T.C., Q.C., Q.X., L.Z.); 3 State Key Laboratory of Cell Biology, CAS Center for Excellence in Molecular Cell Science, Institute of Biochemistry and Cell Biology, Shanghai Institutes for Biological Sciences, University of Chinese Academy of Sciences, China (B.Z.).

**Keywords:** arteriosclerosis, lineage tracing, metabolism, stem cells, transplantation

## Abstract

**Rationale:**

Transplantation-accelerated arteriosclerosis is one of the major challenges for long-term survival of patients with solid organ transplantation. Although stem/progenitor cells have been implicated to participate in this process, the cells of origin and underlying mechanisms have not been fully defined.

**Objective:**

The objective of our study was to investigate the role of c-Kit lineage cells in allograft-induced neointima formation and to explore the mechanisms underlying this process.

**Methods and Results:**

Using an inducible lineage tracing Kit-CreER;Rosa26-tdTomato mouse model, we observed that c-Kit is expressed in multiple cell types in the blood vessels, rather than a specific stem/progenitor cell marker. We performed allograft transplantation between different donor and recipient mice, as well as bone marrow transplantation experiments, demonstrating that recipient c-Kit^+^ cells repopulate neointimal smooth muscle cells (SMCs) and leukocytes, and contribute to neointima formation in an allograft transplantation model. c-Kit–derived SMCs originate from nonbone marrow tissues, whereas bone marrow-derived c-Kit^+^ cells mainly generate CD45^+^ leukocytes. However, the exact identity of c-Kit lineage cells contributing to neointimal SMCs remains unclear. ACK2 (anti-c-Kit antibody), which specifically binds and blocks c-Kit function, ameliorates allograft-induced arteriosclerosis. Stem cell factor and TGF (transforming growth factor)-β1 levels were significantly increased in blood and neointimal lesions after allograft transplantation, by which stem cell factor facilitated c-Kit^+^ cell migration through the stem cell factor/c-Kit axis and downstream activation of small GTPases, MEK (mitogen-activated protein kinase kinase)/ERK (extracellular signal–regulated kinase)/MLC (myosin light chain), and JNK (c-Jun N-terminal kinase)/c-Jun signaling pathways, whereas TGF-β1 induces c-Kit^+^ cell differentiation into SMCs via HK (hexokinase)-1–dependent metabolic reprogramming and a possible downstream O-GlcNAcylation of myocardin and serum response factor.

**Conclusions:**

Our findings provide evidence that recipient c-Kit lineage cells contribute to vascular remodeling in an allograft transplantation model, in which the stem cell factor/c-Kit axis is responsible for cell migration and HK-1–dependent metabolic reprogramming for SMC differentiation.

## Abstract 2

Supplemental Digital Content is available in the text.

Establishment of organ transplantation gives hope for patients experiencing end-stage of organ failure.^1^ While graft failure and multiple organ dysfunctions are leading causes of death in the early years after transplantation, transplant arteriosclerosis is highly related to poor prognosis and severely limits the long-term survival of patients. Transplantation-accelerated arteriosclerosis is initiated by acute innate and acquired immune responses,^2^ which lead to endothelial cell (EC) damage and dysfunction, followed by smooth muscle cell (SMC) accumulation to form the lesions.^1^ The critical role of SMCs in arteriosclerosis has been extensively established,^3^ but the origin of neointimal SMCs remains controversial.^4^ It was historically believed that medial SMCs undergo phenotypic switching, migrate to the intimal region and proliferate to form neointima.^5^ However, neointimal SMCs have been found to be heterogeneous, and recent studies have revealed various origins of SMCs may contribute to neointima formation.^6,7^ In addition to medial SMCs, various cell types including ECs which may undergo endothelial-to-mesenchymal transition, bone marrow-derived or circulating SMC progenitors, fibroblasts as well as vascular resident stem/progenitor cells (SPCs), have been reported to be possible regulators in this process.^8–14^

**Editorial, see p 242**

**Meet the First Author, see p 148**

Genetic lineage tracing has proved to be a powerful tool to track stem cell fate and identify the progeny of specific cell types during embryonic development as well as in disease models.^15^ Based on inducible lineage tracing models, Kramann et al^13^ have shown that adventitia Gli1^+^ mesenchymal stem cell-like cells are important sources of neointimal SMCs in injured arteries, whereas Roostalu et al^16^ revealed that CD146^+^ immature SMC progenitors in arterial branch sites also contribute to neointima formation in a similar wire-injury model, in which they further proposed that neointimal SMCs may arise from adventitial SPCs. Our group previously identified SPCs expressing Sca (stem cell antigen)-1, c-Kit, and CD34 in aortic adventitia.^12^ When expanded in cell culture and seeded on adventitia of a vein graft model, these SPCs differentiated into SMCs and contributed to neointima formation,^12^ suggesting a potential role of these SPCs in vessel remodeling. However, the precise contribution of SPCs to SMC accumulation in transplant arteriosclerotic lesions remains unknown.

In this study, we used Kit-CreER;Rosa26-tdTomato mice, an inducible lineage tracing model, to trace the fate of c-Kit lineage cells in an aortic allograft mouse model. Our results surprisingly showed that c-Kit labels multiple cell types in the aortic wall. c-Kit^+^ cells repopulate neointimal SMCs and contribute to neointimal formation in aortic grafts. The origin of these c-Kit–derived SMCs is from recipient nonbone marrow tissues, whereas bone marrow c-Kit^+^ cells mainly generate leukocytes. Additionally, we confirmed the SMC differentiation potential of isolated c-Kit^+^ cell in vitro. We further investigated possible underlying mechanisms for SMC differentiation from c-Kit^+^ cell, and revealed that stem cell factor (SCF) triggers cell migration through SCF/c-Kit signaling pathway, whereas TGF (transforming growth factor)-β1 induces SMC differentiation via metabolic reprogramming.

## Methods

The data that support the findings of this study are available on reasonable request. All animal experiments performed were approved by the UK Home Office (PPL70/8944).

An additional Detailed Methods is provided in the Online Data Supplement.

## Results

### Characterization of c-Kit^+^ Cells in the Aorta

**Figure 1.**
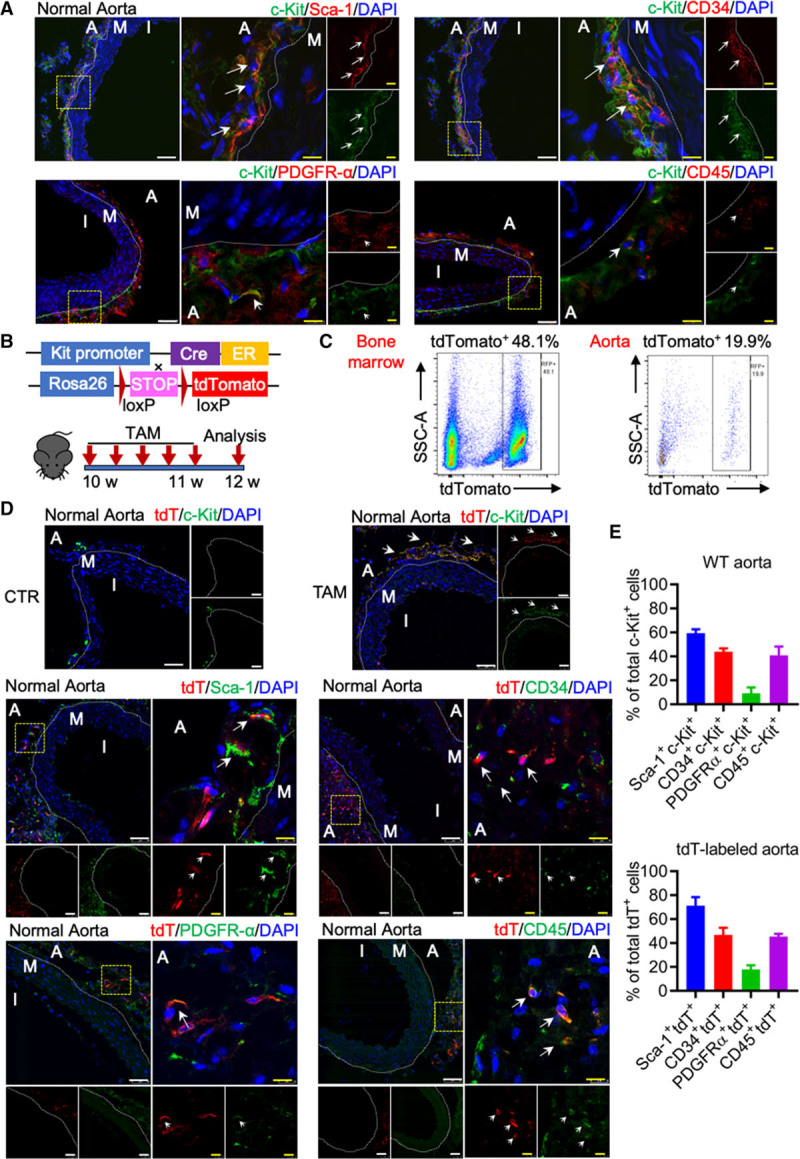
**Characterization of c-Kit+ cells in mouse aorta. A**, Representative images of normal aorta from wild-type (WT) C57BL/6J mice, stained for c-Kit, Sca (stem cell antigen)-1, CD34, PDGFR (platelet-derived growth factor receptor)-α, and CD45 (n=6 per group). Arrows indicate examples of double positive cells. **B**, Strategy for Kit-CreER;Rosa26-tdTomato mice generation and experimental schedule of tamoxifen-induced tdTomato labeling of c-Kit^+^ cells. **C**, Representative flow cytometric analysis of tdTomato^+^ cells in bone marrow and aorta (n=6 per group). **D**, Representative images showing staining of tdTomato, c-Kit, Sca-1, CD34, PDGFR-α, and CD45 in the control (corn oil) or tamoxifen-treated mice (n=6 per group). Arrows indicate examples of co-staining cells. **E**, Quantification of co-staining cells of WT aorta in **A** and tdTomato-labeled aorta in **D**. Data represent mean±SEM, n=3. Scale bars, 50 μm (white) and 10 μm (yellow). A indicates adventitia; CTR, control; I, intima; M, media; TAM, tamoxifen; and tdT, tandem dimer Tomato.

To characterize and establish the distribution of vascular resident c-Kit^+^ cells, we stained aorta isolated from wild-type C57BL/6J mice for c-Kit, together with Sca-1, CD34, PDGFR (platelet-derived growth factor receptor)-α, and CD45. As expected, c-Kit^+^ cells were mainly located in the adventitia (Figure 1A). A majority of c-Kit^+^ cells expressed classical SPC markers Sca-1 (≈60%) and CD34 (≈44%). Only ≈9% of c-Kit^+^ cells co-expressed PDGFR-α, a marker for fibroblast or mesenchymal stem cell. Interestingly, we also identified a large population (≈41%) of c-Kit^+^ CD45^+^ cells (Figure 1A and 1E), which could be leukocytes. However, considering that most mature immune cells do not express c-Kit,^17^ this population may also be a subpopulation of recently identified adventitial macrophage progenitor cells distinct from circulating leukocytes.^18,19^ Additional en face staining further confirmed distribution of c-Kit^+^ cells co-expressing Sca-1 and CD34 in the aortic adventitia (Online Figure IA). To further address the characterization of c-Kit^+^ cells, a Kit-CreER;Rosa26-tdTomato mouse was used.^20,21^ tdTomato (tandem dimer Tomato) specifically labels c-Kit^+^ cells upon tamoxifen injection. Kit-CreER;Rosa26-tdTomato mice were pulsed with tamoxifen and analyzed for tdTomato expression one week later (Figure 1B). Flow cytometric analysis showed successful labeling of tdTomato in bone marrow, aorta, and other established c-Kit expressing tissues (Figure 1C; Online Figure II). We also observed cells co-expressing both tdTomato and c-Kit in the aortic adventitia (Figure 1D). Consistent with our results from wild-type mice, a similar proportion of tdTomato^+^ cells expressing Sca-1 (≈71%), CD34 (≈47%), PDGFR-α (≈18%), and CD45 (≈46%) were observed (Figure 1D and 1E; Online Figure III). Specifically, ≈30% of the tdTomato^+^ cells were Sca-1^+^ CD34^+^ (Online Figure III). tdTomato was rarely detected in the media, however, we did observe a minor population of medial SMCs labeled by tdTomato, representing ≈0.1% of total medial SMCs (Online Figure IV). These data collectively indicate that, although c-Kit was previously thought to be a specific SPC marker and did label a population of previously identified SPCs in the aorta, vascular c-Kit^+^ cells actually turn out to be a heterogeneous population. Besides, a c-Kit^+^ population expressing Sca-1 and CD34, a possible subpopulation of hematopoietic stem cells, was also observed in the bone marrow (Online Figure IB).

### Recipient c-Kit^+^ Cells Repopulate SMCs in the Neointima of Allograft Model

We further tested whether c-Kit^+^ cells may contribute to neointima formation during vascular remodeling. We used a transplant arteriosclerosis mouse model as previously described,^22^ in which donor aortic segments from BALB/c mice were transplanted into recipient C57BL/6J mice (Online Figure VA). We^23^ and others^24–26^ have previously demonstrated that transplant arteriosclerosis is a severe vascular injury model caused by alloimmune reaction, which may possibly induce cell apoptosis in all vascular layers (ECs, SMCs, or even adventitial cells). Immunostaining showed neointima formation after allograft transplantation (Online Figure VB). Importantly, c-Kit^+^ cells expressing Sca-1 and CD34 were found in the neointima, with a significantly higher number compared to normal aorta (Online Figure VC), suggesting a possible role of c-Kit^+^ SPCs in allograft-induced neointima formation.

**Figure 2.**
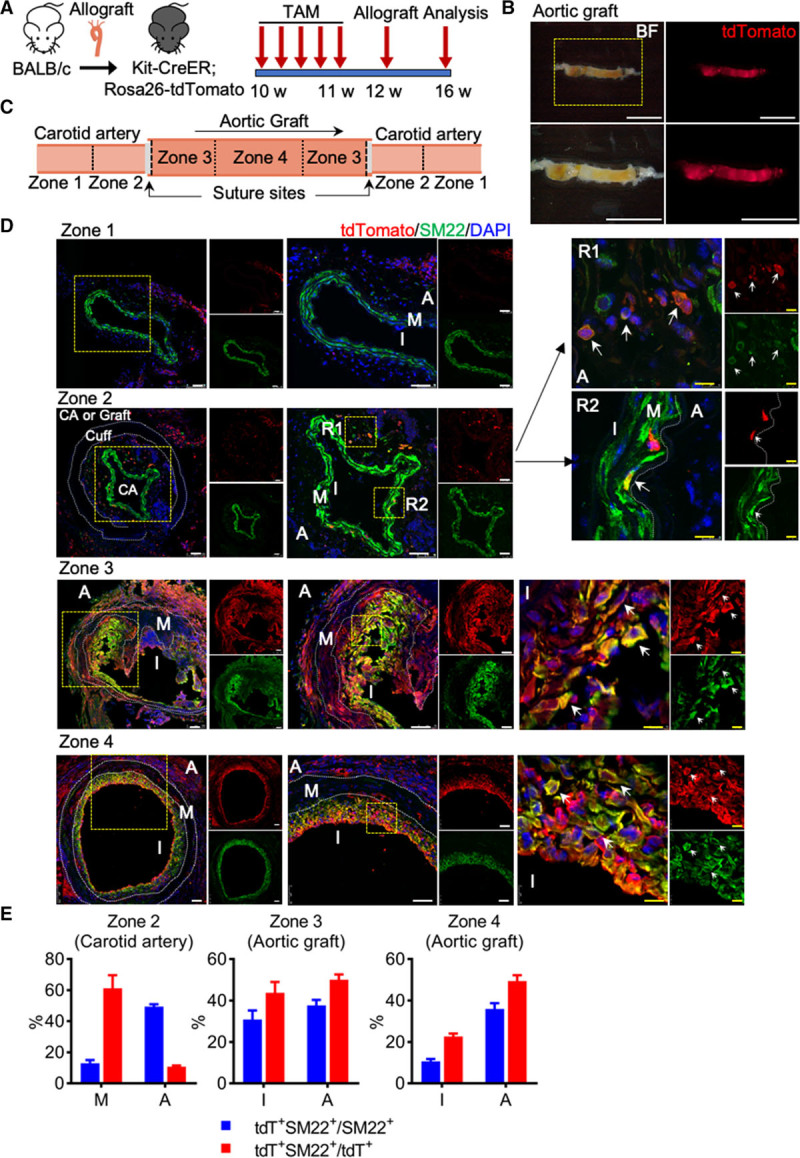
**Recipient c-Kit+ cells repopulate smooth muscle cells (SMCs) in transplant arteriosclerosis. A**, Schematic showing procedure for allograft transplantation experiments. After Kit-CreER;Rosa26-tdTomato mice received tamoxifen injection, aortic segments from BALB/c mice were transplanted into these mice and grafts were collected 4 wk after surgery (n=6 per group). **B**, Representative whole-mount images showing tdTomato labeling in aortic grafts with adjacent tissues (n=3). Scale bars: 5 mm. **C**, Schematic showing distinct zones of aortic graft with adjacent carotid arteries. Zone 1 is carotid artery that lies distal from the site of anastomosis. Zone 2 is carotid artery proximal to the site of anastomosis and encompasses the suture site. Zone 3 and zone 4 are aortic grafts proximal to, or distal from the site of anastomosis, respectively. **D**, Representative images showing different zones of aortic allograft with adjacent carotid arteries, stained with tdTomato and SM22 (smooth muscle protein 22). Arrows indicate tdTomato^+^SM22^+^ cells. Scale bars, 50 μm (white) and 10 μm (yellow). **E**, Graphs showing quantification of tdTomato expression in SM22^+^ SMCs or SM22 expression in tdTomato^+^ cells. Data represent mean±SEM, n=6 in zone 2, n=12 in zone 3 and zone 4. A indicates adventitia; CA, carotid artery; I, intima or neointima; M, media; R1-2, region 1-2; and tdT, tandem dimer Tomato.

Genetic lineage tracing of c-Kit^+^ cells was next performed using Kit-CreER;Rosa26-tdTomato mice. Mice pulsed with tamoxifen were subjected to an allograft transplantation surgery, in which aorta segments from BALB/c mice were transplanted into Kit-CreER;Rosa26-tdTomato mice. Grafts with adjacent carotid arteries were collected 4 weeks later for analyses (Figure 2A). Whole-mount fluorescence images showed that aortic graft (from donor wild-type BALB/c mice) was strongly labeled by tdTomato, with a relatively even distribution across the whole graft (Figure 2B). Immunostaining confirmed the abundant accumulation of tdTomato^+^ cells in the neointimal lesions (Online Figure VI), indicating that c-Kit^+^ cells may participate in allograft-induced neointima formation. As SMCs are the major component of the neointima, we further explored whether c-Kit^+^ cells give rise to neointimal SMCs. Grafts with adjacent tissues were divided into different zones for analyses (Figure 2C). Carotid artery from zone 1, which lies distal from the site of anastomosis, showed normal and similar morphology as healthy artery, with SM22^+^ (smooth muscle protein 22) SMCs detected in the media and tdTomato^+^ cells in the adventitia (Figure 2D). In zone 2, where carotid artery is proximal to the site of anastomosis and encompasses the suture site (consisting of carotid artery, cuff, and graft), a large number of tdTomato^+^ cells were detected in the carotid artery, a connecting region between the carotid artery and a region outside the cuff (Figure 2D). This region outside the cuff could be some parts of carotid artery and allograft. A small population of SM22^+^tdTomato^+^ cells was also observed in both medial and adventitial layers (Figure 2D and 2E). Of note, the labeling of SMC by c-Kit is variable in different regions of the adjacent carotid artery, with a much higher percentage (≈12.9%) of medial tdTomato^+^ SMC in zone 2, compared to a lower percentage (≈0.1%) in carotid artery distant to the graft (zone 1). Zone 3 and zone 4 are aortic grafts proximal to, or distal from the site of anastomosis, respectively (Figure 2C). While zone 3 formed a more severe and irregular neointima, zone 4 formed a more organized neointima (Figure 2D). SM22^+^tdTomato^+^ cells were detected in both zones, with 30.9±4.4% of neointimal SM22^+^ SMCs in zone 3, and 10.6±1.2% in zone 4 labeled by tdTomato (Figure 2D and 2E). Notably, the labeling of total neointimal SMCs by tdTomato is highly variable even in the same zone of the aortic grafts, ranging from 6.2% to 55.4% in zone 3, and from 5.2% to 18.0% in zone 4 (Figure 2D and 2E; Online Figure VII). These data suggest that c-Kit^+^ cells repopulate neointimal SMCs. Furthermore, the higher proportion of c-Kit–derived neointimal SMCs in zone 3 than zone 4, and the seemingly expansion of c-Kit^+^ cells in carotid artery (zone 2), suggest a possibility that a specific population of c-Kit^+^ cells may migrate from adjacent carotid artery to the graft and generate neointimal SMCs. Interestingly, a population of SM22^+^tdTomato^+^ cells was also observed in the adventitia of the aortic graft, with a similar proportion in both zone 3 and 4 (Figure 2D and 2E; Online Figure VII). Single cell suspensions were also obtained from aortic grafts and analyzed by immunostaining and flow cytometry, further confirming the existence of c-Kit–derived SMCs (Online Figure VIII). Collectively, these data suggest c-Kit^+^ cells give rise to neointimal SMCs. As tdTomato only labeled recipient cells in this model, our results also indicate that recipient c-Kit^+^ cells repopulate neointimal SMCs.

To further investigate whether neointimal SMCs may arise from c-Kit^+^ cells within a donor graft, tdTomato-labeled aortic segments from Kit-CreER;Rosa26-tdTomato mice were transplanted into BALB/c mice, and grafts were collected for analysis 4 weeks later (Online Figure XA). Successful labeling of tdTomato in the aorta before transplantation was confirmed by whole-mount fluorescence microscopy (Online Figure XB, left). Interestingly, although we observed neointimal SMC accumulation in aortic grafts, no tdTomato^+^ cells were detected by both whole-mount fluorescence view and immunostaining sections (Online Figure XB and XC). Collectively, these results demonstrate that c-Kit^+^ cells from recipient, but not donor mice, are essential contributors to neointimal SMC accumulation in an allograft model.

### Nonbone Marrow Source of c-Kit^+^ Cells Differentiate into Neointimal SMCs, While Bone Marrow c-Kit^+^ Cells Give Rise to CD45^+^ Leukocytes in Transplant Arteriosclerosis

**Figure 3.**
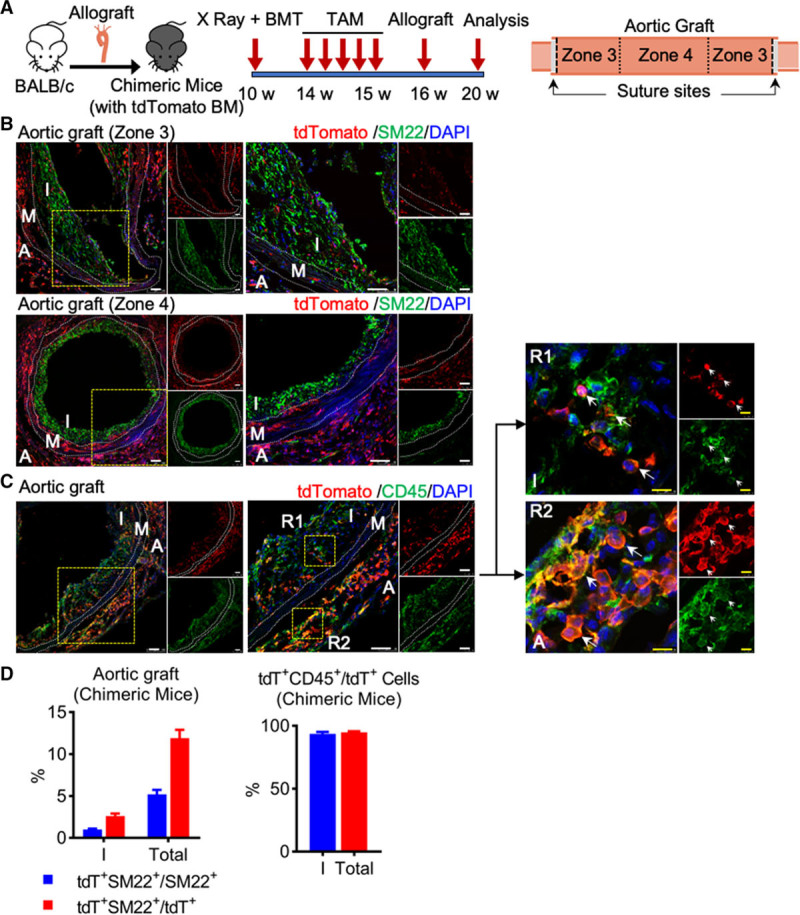
**Nonbone marrow source of c-Kit+ cells repopulates neointimal smooth muscle cells (SMCs), whereas bone marrow c-Kit+ cells give rise to CD45+ leukocytes in transplant arteriosclerosis. A**, Strategy for generation of chimeric mouse model, in which bone marrow cells from Kit-CreER;Rosa26-tdTomato mice were transplanted into irradiated C57BL/6J mice, followed by tamoxifen treatment and allograft transplantation (n=6 mice per group). Aortic grafts were divided into different zones for analyses. **B**, Representative immunostaining images showing staining of tdTomato and SM22 (smooth muscle protein 22) in aortic grafts of chimeric mice. **C**, Representative immunostaining images showing staining of tdTomato and CD45 in aortic grafts of chimeric mice. Arrows indicate double positive cells. **D**, Quantification of tdTomato^+^, SM22^+^, and CD45^+^ cells in neointima or the whole graft. Data represent mean±SEM, n=6 per group. Scale bars, 50 μm (white) and 10 μm (yellow). A indicates adventitia; I, neointima; M, media; R1-2, region 1-2; and tdT, tandem dimer Tomato.

We next sought to address whether recipient c-Kit^+^ cells originate from bone marrow or nonbone marrow tissues. Chimeric mice were generated, in which bone marrow cells from Kit-CreER;Rosa26-tdTomato mice were transferred to irradiated wild-type C57BL/6J mice, and further pulsed with tamoxifen and subjected to allograft transplantation (Figure 3A). Successful reconstitution of bone marrow from tdTomato mice was confirmed by the detection of tdTomato^+^ cells in bone marrow, peripheral blood, as well as aortic graft (Online Figure XI; Figure 3B). Although tdTomato^+^ cells were detected in neointimal lesions, only ≈1% neointimal SM22^+^ cells were tdTomato^+^ (Figure 3B and 3D), suggesting the origin of most c-Kit–derived SMCs was nonbone marrow tissues.

Although most bone marrow c-Kit–derived cells do not produce SMCs, this population may also play an important role in allograft-induced neointima formation. Considering that transplant arteriosclerosis is mediated by alloimmune reactions, we supposed that this bone marrow c-Kit–derived population could be CD45^+^ leukocytes. CD45 staining was further performed in both chimeric model and the allograft model from Figure 2. Our results showed that around half of tdTomato^+^ cells were positive for CD45 in the allograft model from Figure 2 (Online Figure IXA and IXB), whereas most (≈95%) tdTomato^+^ cells expressed CD45 in this chimeric model (Figure 3C and 3D). Of note, tdTomato^−^CD45^+^ cells were also observed in allograft from the chimeric model (Figure 3C), suggesting that not all CD45^+^ cells are derived from bone marrow c-Kit lineage cells. Our data further showed that most tdTomato^+^CD45^+^ cells and tdTomato^+^SM22^+^ cells were separate populations (Online Figure IXC). Thus, bone marrow-derived c-Kit^+^ cells mainly generate CD45^+^ leukocytes, rather than SMCs, and may also be an important contributor to neointima formation.

### Blocking of c-Kit by ACK2 Significantly Ameliorates Allograft-Induced Neointima Formation

**Figure 4.**
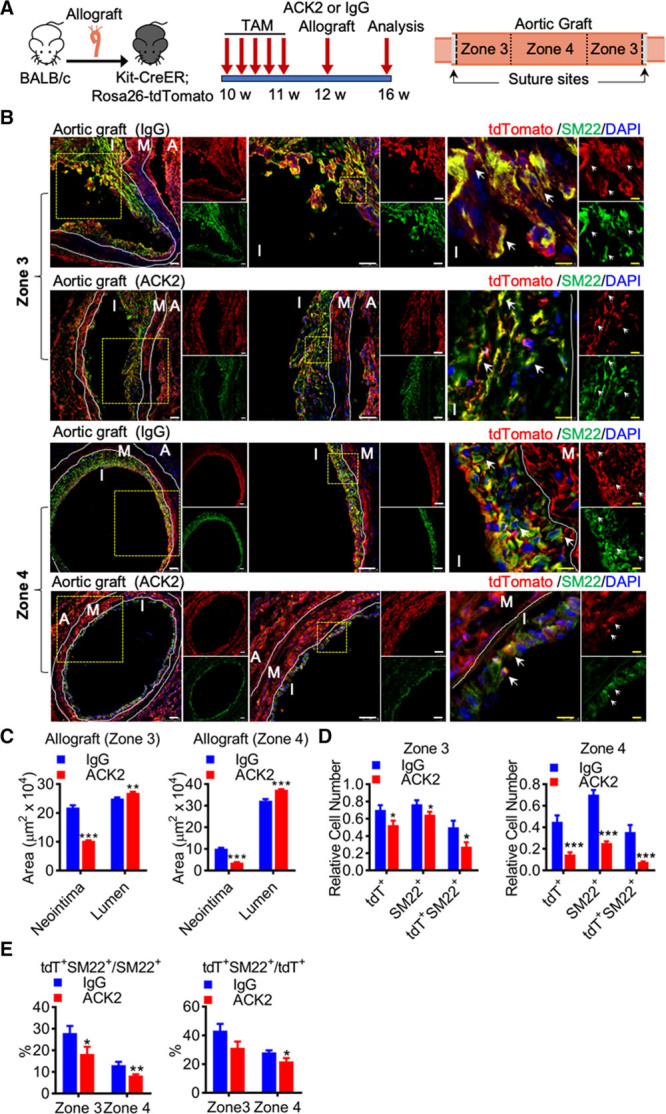
**Blocking of c-Kit+ cells by ACK2 (anti-c-Kit antibody) ameliorates transplant arteriosclerosis. A**, Schematic showing ACK2 or control IgG treatment in mouse allograft model (n=6 mice per group). Aortic grafts were divided into different zones for analyses. **B**, Representative images showing staining of tdTomato and SM22 (smooth muscle protein 22) in zone 3 and zone 4 of the aortic graft from control IgG or ACK2-treated mice. Arrows indicate examples of co-staining cells. Scale bars, 50 μm (white) and 10 μm (yellow). **C**, Graphs showing quantification of neointimal and luminal areas. **D**, Graph showing quantification of relative cell number of tdTomato^+^ cells and SM22^+^ cells in the neointima. **E**, Graphs showing percentage of tdTomato^+^ cells in smooth muscle cells (SMCs) and SM22^+^ cells in total tdTomato^+^ cells in the neointima. Data represent mean±SEM (n=5 in **C**, n=12 in **D** and **E**). *P<0.05, **P<0.01, and ***P<0.001 by unpaired 2-tailed *t* test (**C**–**E**). A indicates adventitia; I, neointima; M, media; and tdT, tandem dimer Tomato.

Data so far suggested that recipient nonbone marrow c-Kit^+^ cells produce SMCs, whereas bone marrow c-Kit^+^ cells generate leukocytes. We next asked whether blocking of c-Kit could abrogate neointima formation in our model. ACK2 (anti-c-Kit antibody), a monoclonal antibody reacting against the extracellular domain of c-Kit, has been reported to specifically target c-Kit^+^ cells and block c-Kit function,^27^ and was further used in our experiments. Kit-CreER;Rosa26-tdTomato mice pulsed with tamoxifen were subjected to allograft transplantation (Figure 4A). Immediately after transplantation, ACK2 or control IgG mixed with Pluronic F-127 Gel was applied to the adventitial side of the grafts, which allows a slow release of ACK2 to the local graft microenvironment.^28^ Four weeks later, graft tissues were harvested for analyses (Figure 4A). Whole-mount fluorescence analyses showed a significant decrease of tdTomato in ACK2 group compared to IgG group (Online Figure XII), indicating that ACK2 markedly reduced accumulation of c-Kit–derived cells in the graft. ACK2 reduced neointima formation in both zone 3 (≈53%) and zone 4 (≈64%), as well as accumulation of neointimal tdTomato^+^ cells (decreased by ≈25% in zone 3 and ≈68% in zone 4) and SM22^+^ cells (decreased by ≈16% in zone 3 and ≈64% in zone 4; Figure 4B and 4D). More importantly, the number of neointimal tdTomato^+^ SM22^+^ cells was also downregulated in ACK2 group, by ≈45% in zone 3 and ≈78% in zone 4 (Figure 4B and 4D). Staining of α-SMA (smooth muscle actin) and calponin was also performed and showed similar results (Online Figure XIII). The percentage of tdTomato^+^ SM22^+^ cells in total SM22^+^ cells was lower in ACK2 group in both zones (Figure 4E, left). While the percentage of tdTomato^+^ SM22^+^ cells in total tdTomato^+^ cells was reduced by 28% (although not significant, *P*=0.07) in zone 3, it was significantly decreased by ≈22% in zone 4 (Figure 4E, right). Notably, variable co-staining of SM22 and tdTomato was also observed in different sections (Figure 4B and 4E; Online Figure XIV). Although we cannot exclude the possibility that ACK2 may also impair neointima formation via other unknown effects, these data provide clear evidence to support the critical role of c-Kit^+^ cells in neointimal formation of allograft mouse model.

### SCF Induces c-Kit^+^ Cell Migration

**Figure 5.**
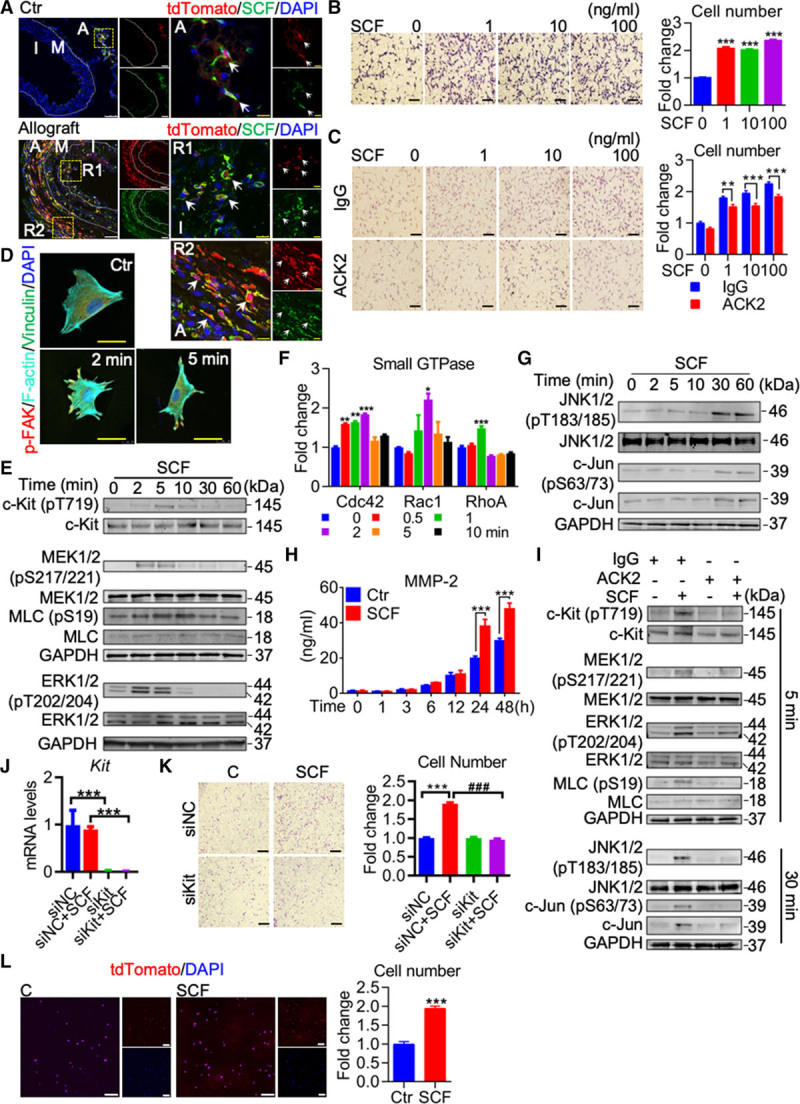
**Stem cell factor (SCF) induces migration of c-Kit+ cells in vitro. A**, Representative images showing tdTomato and SCF staining in control aorta from Kit-CreER;Rosa26-tdTomato mice described in Figure 1B, and aortic allografts from mouse model described in Figure 2A (n=6 per group). Arrows indicate co-staining of tdTomato and SCF. **B** and **C**, Representative images showing SCF-induced c-Kit^+^ cell migration (**B**), with or without ACK2 (anti-c-Kit antibody) or control IgG (**C**) by transwell migration assay. Graphs shown are relative cell number normalized to control. n=30 (10 random fields per experiment and 3 independent experiments) in **B**, n=15 (5 random fields per experiment and 3 independent experiments) in **C**. **D**, Representative images showing cell morphology of SCF-treated c-Kit^+^ cells stained with p-FAK (phosphorylated focal adhesion kinase), F-actin, and vinculin (n=3). **E**, Representative Western blot showing activation of c-Kit, MEK (mitogen-activated protein kinase kinase)-ERK (extracellular signal-regulated kinase)-MLC (myosin light chain) pathways in response to SCF (n=3). **F**, Graphs showing activation of small GTPase including Cdc42 (cell division cycle 42), Rac1 (Rac family small GTPase 1), and RhoA (Ras homolog family member A) in SCF-treated c-Kit^+^ cells (n=3). **G**, Representative Western blot indicating activation of JNK (c-Jun N-terminal kinase)/c-Jun pathways in response to SCF (n=3). **H**, Quantification of MMP (matrix metalloproteinase)-2 in cell culture supernatant from SCF-treated c-Kit^+^ cells (n=3). **I**, Representative Western blot showing signaling pathways in response to SCF for indicated times, in the presence of ACK2 or IgG (n=3). **J** and **K**, c-Kit^**+**^ stem/progenitor cells (SPCs) were transfected with negative control (NC) or Kit siRNA and further treated with or without SCF. **J**, mRNA expression of Kit in different groups were measured to show the efficiency of siRNA knockdown (n=4). **K**, Transwell migration assay was used to determine cell mobility (n=5). **L**, tdTomato-labeled c-Kit^**+**^ cells were isolated from Kit-CreER;Rosa26-tdTomato mice to test cell migration in response to SCF. Images showed migrating cells on the transwell filter stained with tdTomato and DAPI (n=5). Scale bars, black (100 μm in **B**, **C**, and **K**), white (50 μm in **A** and **L**), and yellow (10 μm in **A** and **D**). All data shown are mean±SEM. *P<0.05, **P<0.01, ***P<0.001, ###*P*<0.001, by unpaired 2-tailed *t* test (**L**), 1-way ANOVA with Dunnett test (**B** and **F**), 1-way ANOVA with Tukey test (**J** and **K**), and 2-way ANOVA with Bonferroni test (**C** and **H**). A indicates adventitia; I, intima or neointima; M, media; R1-2, region 1-2; and tdTomato, tandem dimer Tomato.

Previous reports have shown that SCF, a specific ligand for c-Kit, can mediate cell survival and proliferation as well as SMC migration.^17^ To examine the possible mechanisms underlying c-Kit^+^ cell migration to the lesions and subsequent differentiation into neointimal SMC, SCF presence was measured in blood and the vessel wall of allograft models. A significant increase in SCF concentrations in peripheral blood was observed after allograft transplantation (Online Figure XVA). Compared to control aorta, significant increases of both SCF and tdTomato were detected and found to be colocalized in the allograft (Figure 5A), suggesting a possibility that increased accumulation of SCF may induce migration of c-Kit^+^ cells to the lesion sites. Control aorta from donor BALB/c mice, and donor aortic grafts one day after allograft transplantation were also analyzed and showed that SCF was markedly increased in the adventitia of aortic graft only one day after transplantation (Online Figure XVI). More importantly, accumulation of recipient tdTomato^+^ cells was detected in the adventitia, where SCF was highly expressed (Online Figure XVI), further supporting that SCF may induce c-Kit^+^ cell migration.

To further validate our hypothesis, we isolated c-Kit^+^ cells from these grafts from wild-type mice as described previously.^12^ Phenotypic analyses by flow cytometry showed that most isolated c-Kit^+^ cells expressed classical stem/progenitor markers including Sca-1 and CD34, fibroblast or mesenchymal stem cell marker CD140a (PDGFR-α), but not leukocyte marker CD45 or SMC marker α-SMA (Online Figure XVII). Moreover, these isolated c-Kit^+^ cells can be cultured for over 30 passages without significant changes in vitro (data not shown), suggesting that these isolated c-Kit^+^ cells displayed stem/progenitor properties and may represent a population of SPC and fibroblast in vivo. We next determined whether SCF could induce migration of these isolated c-Kit^+^ cells in vitro. Both transwell migration assay and scratch wound healing assay showed that SCF significantly increased migration of c-Kit^+^ cells in a dose-dependent manner (Figure 5B; Online Figure XVIIIA). As blocking of c-Kit by ACK2 could markedly reduce neointima formation in vivo, we determined if ACK2 could also reduce SCF-induced c-Kit^+^ cell migration in vitro. As expected, ACK2-treated cells showed a significantly lower migration rate than a control IgG group in response to SCF stimulation (Figure 5C), without affecting cell proliferation in c-Kit^+^ cells (Online Figure XVIIIB and XVIIIC). Taken together, our data provide evidence that SCF is a chemotactic factor that induces c-Kit^+^ cell migration in vitro.

### Activation of SCF/c-Kit Signaling Pathway Regulates c-Kit^+^ Cell Migration

We next investigated the underlying mechanisms behind SCF-mediated cell migration. Considering that reorganization of the actin cytoskeleton is critical in triggering cell migration,^29^ we first performed immunostaining to observe the actin cytoskeleton and focal adhesions in c-Kit^+^ cells, using fluorescent-phalloidin, which specifically binds actin, and antibodies against p-FAK (phosphorylated focal adhesion kinase) and vinculin to stain focal adhesion assembly. SCF immediately induced formation of parallel elongated-stress fibers and cell spreading in c-Kit^+^ cells, compared to untreated cells (Figure 5D). Filopodia and lamellipodia were formed at the leading edge of c-Kit^+^ cells within 2 minutes of SCF treatment. Moreover, co-staining of p-FAK, vinculin, and F-actin was also observed at the leading edge (Figure 5D), which is an indicative of cell migration. These data suggest that SCF may trigger cell migration at a very early time point. As SCF has been reported to specifically bind and activate c-Kit, we sought to investigate whether c-Kit signaling pathways regulate this early process. Phosphorylation of c-Kit was increased by SCF within minutes (Figure 5E, upper), indicating that early activation of c-Kit signaling may regulate this process. Small GTPases, which can be activated by tyrosine kinase, have been shown to be pivotal in regulating cytoskeleton reorganization and cell migration.^29^ As c-Kit is a receptor tyrosine kinase, we performed a G-LISA activation assay to test the activation of GTPases. We showed that SCF treatment led to early activation of small GTPases including Cdc42 (cell division cycle 42), RhoA (Ras homolog family member A), and Rac1 (Rac family small GTPase 1) within 2 minutes (Figure 5F). Possible downstream pathways of c-Kit signaling and GTPases including phosphorylation of MEK1/2 (mitogen-activated protein kinase kinase 1/2), ERK1/2 (extracellular signal–regulated kinase 1/2), and MLC (myosin light chain) were also elevated shortly after SCF stimulation (Figure 5E, lower). Activation of MLC may further promote cell contractility and cell motility.^30^ Phosphorylation of JNK (c-Jun N-terminal kinase) and c-Jun was also increased at a later time point of 30 minutes (Figure 5G). Because both MEK/ERK and JNK/c-Jun have been reported to regulate expression of MMPs (matrix metalloproteinases) to degrade extracellular matrix and ease cell migration,^31^ we further measured secretion of MMPs in cell culture supernatants. A significant increase in MMP-2, but not MMP-9, was detected after SCF treatment for 24 and 48 hours (Figure 5H; Online Figure XVIIID), suggesting a possible role of MMP-2 in regulating cell migration at a later time. Notably, ACK2 could completely or at least partially block the activation of c-Kit signaling, as well as migration-related pathways including the MEK/ERK/MLC, JNK/c-Jun pathways (Figure 5I). siRNA-mediated knockdown of *Kit* effectively reversed SCF-induced cell migration (Figure 5J and 5K). These results collectively indicate that SCF/c-Kit signaling, upstream of MEK/ERK and JNK pathways, is critical for cell migration in c-Kit^+^ cells. tdTomato-labeled c-Kit^+^ cells were also isolated from the grafts of Kit-CreER;Rosa26-tdTomato mouse using the same method. Flow cytometric analysis confirmed most isolated c-Kit^+^ cells were tdTomato positive (Online Figure XIX). Consistent with our results obtained from nonlabeled c-Kit^+^ cells, SCF also increased migration of tdTomato-labeled c-Kit^+^ cells in vitro (Figure 5L).

### TGF-β1 Induces c-Kit^+^ Cell Differentiation Into SMCs

**Figure 6.**
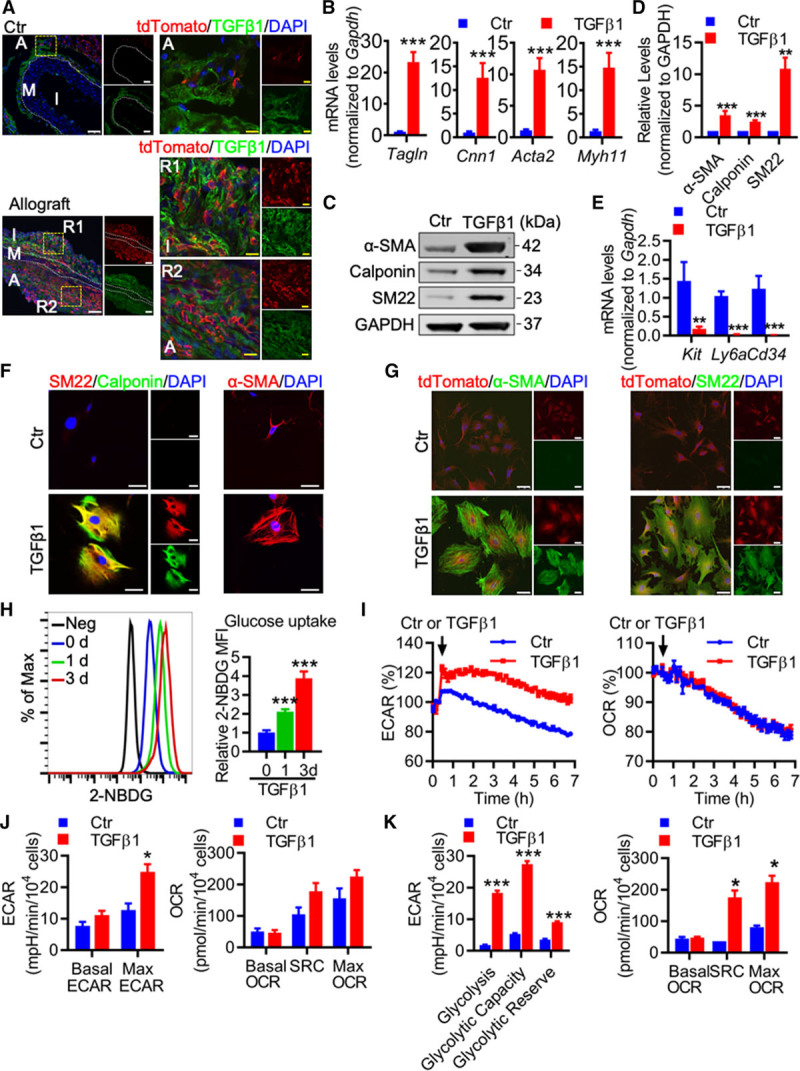
**TGF (transforming growth factor)-β1 induces c-Kit+ cell differentiation into smooth muscle cell (SMC), accompanied by metabolic reprogramming. A**, Representative images showing control aorta from Kit-CreER;Rosa26-tdTomato mice described in Figure 1B, and aortic allografts from mouse model described in Figure 2A stained with tdTomato and TGF-β1 (n=6 per group). **B**–**F**, c-Kit^+^ cells were treated with TGF-β1 (2 ng/mL) for 3 d to induce cell differentiation. **B**, qPCR (quantitative polymerase chain reaction) analyses showing gene expression of smooth muscle markers in TGF-β1–treated c-Kit^+^ cells (n=10). **C** and **D**, Representative Western blot and quantification of smooth muscle markers (n=7). **E**, qPCR analyses of mRNA expression of stem/progenitor markers (n=7 or 10). **F**, Representative images showing staining of smooth muscle markers in c-Kit^+^ cells treated with TGF-β1 (n=3). **G**, tdTomato-labeled c-Kit^+^ cells were isolated from Kit-CreER;Rosa26-tdTomato mice to test cell differentiation in response to TGF-β1 for 3 d in vitro. Representative images showing staining of smooth muscle markers in tdTomato-labeled c-Kit^+^ cells treated with TGF-β1 (n=3). **H**, Representative histogram and quantification of 2-NBDG uptake in c-Kit^**+**^ cells treated with TGF-β1 for indicated times by flow cytometry (n=4). **I**, Real-time monitoring of extracellular acidification rate (ECAR) and oxygen consumption rate (OCR) in c-Kit^**+**^ cells treated with TGF-β1 within 7 h (n=4). **J** and **K**, c-Kit^**+**^ cells were treated with TGF-β1 for 1 d (**J**) or 3 d (**K**). Quantification of ECAR (n=4 in **J** and n=7 in **K**) and OCR (n=4 in **J** and **K**) are shown. All data shown are mean±SEM. *P<0.05, **P<0.01, ***P<0.001, by unpaired 2-tailed *t* test (**B**, **D**, **E**, **J**, and **K**), or 1-way ANOVA with Dunnett test (**H**). Scale bars, 50 μm (white) and 10 μm (yellow). A indicates adventitia; Ctr, control; I, intima or neointima; M, media; Neg, negative control; R1-2, region 1-2; SMA, smooth muscle actin; and tdT, tandem dimer Tomato.

Our data so far suggested that SCF triggers c-Kit^+^ cell migration in vitro, we next investigated whether SCF could also induce c-Kit^+^ cell differentiation into SMC, as most isolated c-Kit^+^ cells do not express SMC marker α-SMA (Online Figure XVII). Our results showed that SCF did not affect expression of SMC markers (Online Figure XXA and XXB). TGF-β1 has been reported to increase in human primary atherosclerotic and restenotic arteries,^32^ as well as various mouse vascular disease models.^8,33,34^ We found that TGF-β1 was also significantly increased in the blood plasma of allograft mice (Online Figure XVB). Moreover, immunostaining showed increased expression of TGF-β1 in the allograft compared to aorta from control mice (Figure 6A), indicating a possible functional role of TGF-β1. To test this hypothesis, we treated c-Kit^+^ cells with TGF-β1 and measured SMC markers in vitro. Both gene and protein expression of SMC markers were markedly increased in c-Kit^+^ cells exposed to TGF-β1 (Figure 6B and 6D), which was also confirmed by immunostaining (Figure 5F). While TGF-β1 upregulated SMC markers in these cells, stem/progenitor markers including *Kit*, lymphocyte antigen 6 complex, locus A (*Ly6a*), and *Cd34* were significantly decreased (Figure 6E), suggesting that c-Kit^+^ cells would downregulate stem/progenitor markers once differentiated. Cell migration was also tested and we showed that c-Kit^+^ cell migration was not affected by low doses of TGF-β1 but was downregulated by higher doses of TGF-β1 (Online Figure XXC). Besides, tdTomato-labeled c-Kit^+^ cells treated with TGF-β1 also showed similar SMC differentiation capacity, as shown by the increased expression of SMC markers in immunostaining images (Figure 6G). Taken together, these data suggest that TGF-β1 can induce c-Kit^+^ cell differentiation into SMC in vitro.

### Glucose Metabolism Is Critical for TGF-β1–Induced c-Kit^+^ Cell Differentiation Into SMC

We further investigated the mechanisms underlying TGF-β1–induced c-Kit^+^ cell differentiation into SMC. Accumulating evidence has demonstrated that cellular metabolism plays a vital role in regulating cell proliferation, differentiation, and function in various cell types.^35–37^ A recent report revealed that satellite cells, the adult resident skeletal muscle stem cells, switched cellular metabolism to glycolysis to activate muscle gene expression and cell differentiation,^38^ suggesting that cellular metabolism may regulate resident stem cell differentiation. We first examined glucose metabolism during TGF-β1–induced c-Kit^+^ cell differentiation. 2-NBDG assay showed that glucose uptake was significantly increased in c-Kit^+^ cells in a time-dependent manner (Figure 6H). Real-time monitoring of extracellular acidification rate (ECAR) and mitochondrial oxygen consumption rate (OCR) was then performed. ECAR is a measure of glycolysis-driven lactate production, whereas OCR indicates mitochondrial oxidative metabolism. Our results showed that TGF-β1 rapidly increased ECAR and this high ECAR level was maintained during 7-hour metabolic monitoring, while OCR remained unchanged (Figure 6I). We then measured metabolism in cells treated with TGF-β1 for 1 and 3 days. c-Kit^+^ cells showed high ECAR level after 1 day (Figure 6J; Online Figure XXIA) and displayed even higher levels in ECAR 3 days later (Figure 6K; Online Figure XXIB), indicating that TGF-β1 dramatically increases glycolysis in c-Kit^+^ cells. No changes were seen in basal OCR after 1 or 3 days. However, we did observe an increasing trend in both spare respiratory capacity and maximal OCR after 1 day, and a significant increase after 3 days (Figure 6J and 6K; Online Figure XXIA and XXIB), suggesting an increased ability to use mitochondrial oxidative metabolism in these cells. Consistent with changes in OCR, mitochondrial mass was also observed to be slightly increased after 1 day, and markedly upregulated after 3 days of TGF-β1 treatment (Online Figure XXIC).

**Figure 7.**
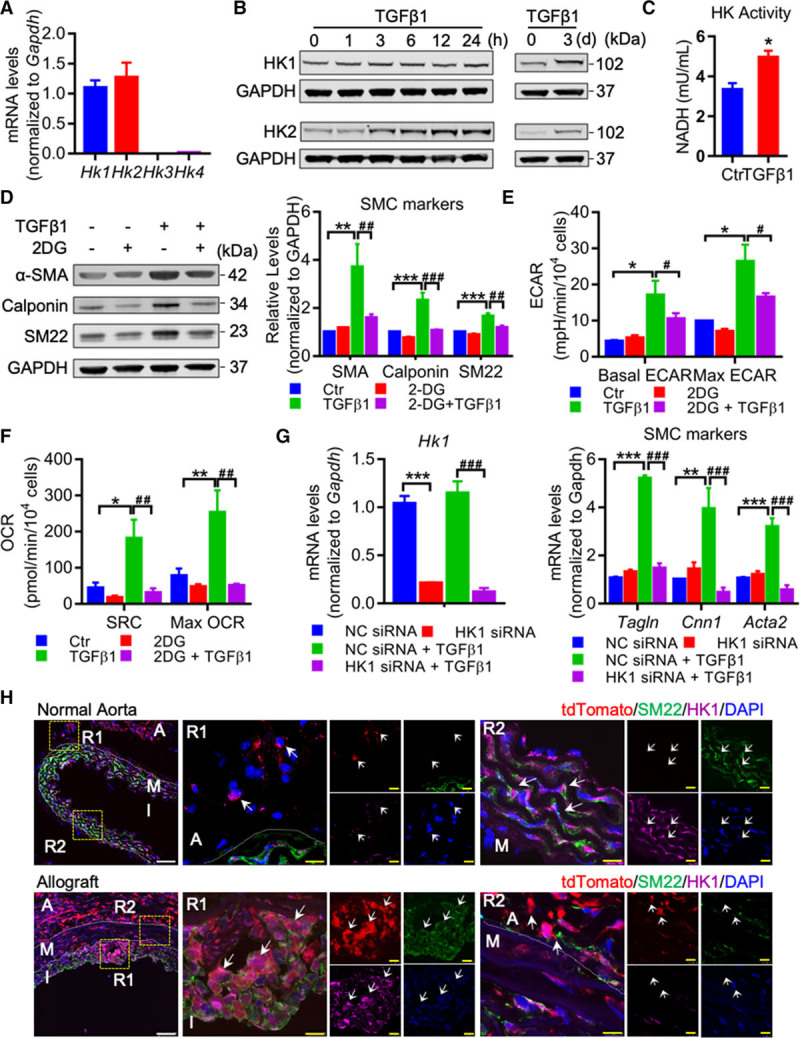
**Glucose metabolism is critical for TGF (transforming growth factor)-β1–induced c-Kit+ cell differentiation into smooth muscle cell (SMC). A**, qPCR (quantitative polymerase chain reaction) analyses for mRNA expression of 4 HK (hexokinase) isozymes in c-Kit^**+**^ cells (n=8 per group). **B** and **C**, c-Kit^**+**^ cells were treated with TGF-β1 for indicated times. **B**, Representative Western blot analyses of HK-1 and HK-2 in TGF-β1–treated c-Kit^**+**^ cells (n=3). **C**, HK activity in TGF-β1–treated cells for 24 h were analyzed (n=4 per group). **D**–**F**, c-Kit^**+**^ cells were treated with TGF-β1 and 2-deoxyglucose (2-DG; 1 mmol/L) for 48 h. Representative Western blot analyses and quantification (**D**) of smooth muscle markers and quantification of extracellular acidification rate (ECAR; **E**) and oxygen consumption rate (OCR; **F**) were shown. n=4 per group in **D**, n=4–5 per group in **E** and **F**. **G**, qPCR analyses of mRNA expression of *Hk1* and smooth muscle markers in c-Kit^**+**^ cells transfected with NC or HK-1 siRNA, treated with or without TGF-β1 for 48 h (n=4 per group). **H**, Representative images showing staining of HK-1, SM22 (smooth muscle protein 22), and tdTomato in normal aorta (from mouse model in Figure 1B) and allograft sections (from mouse model in Figure 2A). Arrows indicate co-staining cells. Scale bars, 50 μm (white) and 10 μm (yellow). Data represent mean±SEM. *P<0.05, **P<0.01, ***P<0.001, #*P*<0.05, ##*P*<0.01, ###*P*<0.001, by unpaired 2-tailed *t* test (**C**), and 1-way ANOVA with Tukey test (**D**–**G**). A indicates adventitia; Ctr, control; I, intima or neointima; M, media; NC siRNA, negative control siRNA; R1-2, region 1-2; SMA, smooth muscle actin; and tdTomato, tandem dimer Tomato.

We next determined whether increased glucose metabolism and glycolysis are essential for cell differentiation. HK (hexokinase), which converts glucose into glucose-6-phosphate, is a rate-limiting enzyme in glucose metabolism and may play a role in this process. Gene expression of different HK isozymes was tested and results showed that c-Kit^+^ cells mainly expressed *Hk1* and *Hk2* (Figure 7A). Western blot analysis showed that while untreated c-Kit^+^ cells expressed HK-1 and HK-2 protein (0 time point), TGF-β1 significantly increased both HK-1 and HK-2 in a time-dependent manner (Figure 7B). Moreover, HK activity was also upregulated (Figure 7C), implicating activation of HK glycolytic activity. We then used 2-deoxyglucose (2-DG), a glucose analog which can be phosphorylated by HK to form 2-deoxyglucose-6-phosphate and cannot be further metabolized,^39^ to block glucose metabolism and examine cell differentiation. 2-DG effectively reduced TGF-β1–induced SMC markers (Figure 7D). Meanwhile, glycolysis, as shown by basal and max ECAR, was also downregulated in cells treated with both 2-DG and TGF-β1 (Figure 7E). Interestingly, 2-DG also reduced both spare respiratory capacity and max OCR (Figure 7F), indicating that glucose metabolism may contribute to the increased ability to use mitochondrial metabolism in these cells. Furthermore, siRNA-mediated knockdown of *Hk1*, but not *Hk2*, effectively reduced SMC markers increased by TGF-β1 (Figure 7G; Online Figure XXIII). Glycogenolysis could also be a cell-intrinsic source of glucose and contribute to glycolysis.^40^ We examined the expression of different isozymes of PYG (glycogen phosphorylase), a rate-limiting enzyme regulating glycogen breakdown pathway, and showed that *Pygb* is expressed in c-Kit^+^ cells (Online Figure XXIVA). However, blocking of PYG by a selective inhibitor CP-91149 did not affect TGF-β1–increased SMC markers (Online Figure XXIVB). Taken together, our data unravel an essential role of HK-1–dependent glucose metabolism in regulating TGF-β1–induced c-Kit^+^ cell differentiation into SMC in vitro. To test whether HK-1 may be also involved in c-Kit–derived neointimal SMC formation in vivo, HK-1 staining was performed in both normal aorta and allograft sections. In normal aorta, HK-1 was detected in both medial SMCs and adventitial tdTomato^+^ cells, with a higher expression in SMCs than tdTomato^+^ cells (Figure 7H, upper). In the allograft, a population of tdTomato^+^SM22^+^ cells with high HK-1 expression was detected in the neointima, whereas some SM22^−^tdTomato^+^ cells with low HK-1 expression was also observed (Figure 7H, lower). Thus, these immunostaining data might suggest a possible role of HK-1 in regulating SMC differentiation from c-Kit^+^ cell in vivo.

**Figure 8.**
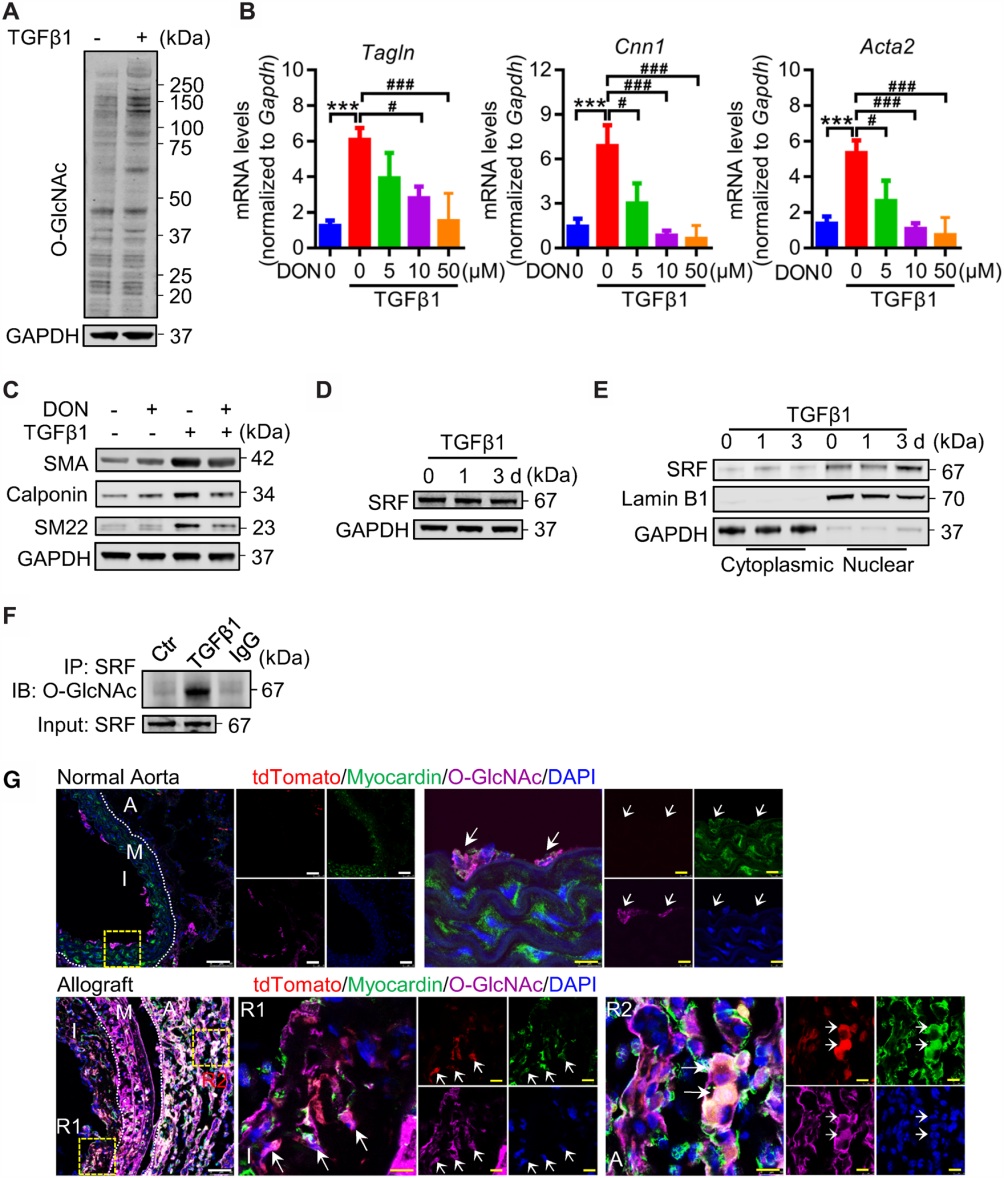
**TGF (transforming growth factor)-β1 induces O-GlcNAcylation of myocardin and serum response factor (SRF**). **A**, Representative Western blot showing protein O-GlcNAcylation in c-Kit^+^ cells after TGF-β1 treatment for 3 d (n=3). **B** and **C**, Quantification of mRNA expression (**B**) and representative Western blot (**C**) showing expression of smooth muscle cell (SMC) markers in TGF-β1–treated c-Kit^+^ cells, with or without 5 μmol/L 6-Diazo-5-oxo-L-norleucine (DON) treatment for 48 h. Data shown are mean±SEM. ***P<0.001, #*P*<0.05, ###*P*<0.001, by 1-way ANOVA with Tukey test, n=7 per group. **D** and **E**, Representative Western blot analysis of SRF in total cell lysates (**D**), and cytoplasmic and nuclear lysates (**E**) of TGF-β1–treated c-Kit^+^ cells for indicated times (n=3). **F**, Representative Western blot showing O-GlcNAcylation of SRF in total cell lysates. SRF in TGF-β1–treated c-Kit^+^ cells for indicated times or 3 d was immunoprecipitated and analyzed for protein O-GlcNAcylation. Total SRF is shown as input (n=3). **G**, Representative images showing staining of myocardin, O-GlcNAc, and tdTomato in normal aorta (from mouse model in Figure 1B) and allograft sections (from mouse model in Figure 2A). Arrows indicate co-staining cells. Scale bars, 50 μm (white) and 10 μm (yellow). A indicates adventitia; I, intima or neointima; M, media; R1-2, region 1-2; SMA, smooth muscle actin; and tdTomato, tandem dimer Tomato.

Growing evidence has suggested an essential role of cellular metabolism in the regulation of epigenetics, including acetylation, methylation, and glycosylation.^41^ The hexosamine biosynthetic pathway, a branch of glucose metabolism, produces UDP-N-acetylglucosamine (UDP-GlcNAc) as a substrate for protein O-GlcNAcylation, which may further modulate protein function.^42^ We then tested whether O-GlcNAcylation was involved in TGF-β1–induced cell differentiation. Our results showed that total protein O-GlcNAcylation was upregulated in TGF-β1–treated c-Kit^+^ cells (Figure 8A). Moreover, both gene and protein analyses revealed that SMC markers increased by TGF-β1 were markedly reversed by 6-Diazo-5-oxo-L-norleucine (DON), an inhibitor of O-GlcNAcylation (Figure 8B and 8C), suggesting a possible role of protein O-GlcNAcylation in regulating cell differentiation. Serum response factor (SRF) are important transcription factors for regulating smooth muscle gene expression.^43^ We showed that TGF-β1 increased nuclear translocation of SRF without affecting total SRF in c-Kit^+^ cells (Figure 8D and 8E). Interestingly, SRF could be modified by O-GlcNAcylation, as O-GlcNAcylation of SRF was significantly increased by TGF-β1 (Figure 8F). Collectively, these data suggest a possible role of O-GlcNAcylation, which is derived from glucose metabolism, in regulating SMC differentiation, and that O-GlcNAcylation of SRF may also participate in this process. O-GlcNAcylation of myocardin was further tested in both normal aorta and allograft sections. In normal aorta, co-staining of myocardin and O-GlcNAc was observed in some medial SMCs underlying the intimal layer, but not in adventitial tdTomato^+^ cells, indicating that O-GlcNAcylation of myocardin may exist in a population of SMCs under physiological conditions (Figure 8G, upper). While tdTomato^+^ myocardin^+^ SMCs were detected in the allograft lesions, these cells were found to co-express O-GlcNAc, suggesting that O-GlcNAcylation of myocardin may be involved in SMC differentiation from c-Kit^+^ cells in vivo.

## Discussion

Arteriosclerosis is characterized by EC dysfunction and thickening of the neointima in which SMCs accumulate.^5^ However, the origin of these neointimal SMCs is still under intense scrutiny.^4^ Recent studies have suggested an important role for resident SPCs in vascular remodeling.^12–14,16^ Multiple SPCs including c-Kit^+^ SPCs have been identified in both mouse and human aortic adventitia and atherosclerotic lesions.^12,44,45^ Previous studies have established that c-Kit is primarily expressed in hematopoietic cells, germ cells, melanocytes, interstitial cells of Cajal, mast cells as well as tissue-resident SPCs.^17^ In this study, we first showed the heterogeneity of adventitial c-Kit^+^ cells in the blood vessels. While a large population expresses classical SPC markers Sca-1 and CD34, other populations including c-Kit^+^ CD45^+^ cells (adventitial macrophage progenitor cell or leukocyte) and c-Kit^+^ PDGFRα^+^ cells (fibroblast or mesenchymal stem cell) were also identified. These data suggest that c-Kit is expressed in multiple cell types. Importantly, c-Kit^+^ cells expressing classical SPC markers Sca-1 and CD34 were also observed within the neointima of aortic grafts, indicating a possible role of c-Kit^+^ SPCs in allograft-induced arteriosclerosis. Using an inducible genetic lineage tracing model, we provided the first evidence that c-Kit^+^ cells are critical contributors of neointimal SMCs in allograft transplantation. Our data that a significant higher proportion of c-Kit–derived SMCs in both sides of the aortic grafts than in the middle part suggest c-Kit lineage cells within the adjacent carotid artery could possibly be an important source of these c-Kit–derived SMCs. Our in vitro data showed that c-Kit^+^ cells isolated from the grafts displayed SPC properties and differentiate into SMCs, further supporting a possible role of c-Kit^+^ SPCs. Thus, c-Kit^+^ SPCs may contribute to neointima formation in our allograft model. However, as c-Kit labels multiple cell types in the vessel wall in our lineage tracing model, the exact identity of c-Kit lineage cells contributing to SMCs still remains unclear.

Based on lineage tracing mouse models, several groups have provided substantial evidence that preexisting SMCs contribute to neointima formation in vascular diseases.^46–52^ Recently, Roostalu et al^16^ revealed that CD146^+^ SMC progenitors contribute to neointimal formation in a mild wire-injury model, but not in a severe transluminal injury model, in which they further suggested adventitial SPCs may play a role. Yuan et al^53^ showed that neointima could be heterogeneous and showed different phenotypes even in the same vascular injury model. While preexisting MYH11^+^ SMCs give rise to most of the neointimal SMCs in one type of neointima, Sox10^+^ SPCs alongside with MYH11^+^ SMCs contribute to form another type.^53^ Thus, the relative contribution of different cell types to neointima formation could be highly context dependent. Chen et al^54^ recently showed that c-Kit^+^ cells rarely contributed to SMCs of neointimal lesions in wire-injured and carotid artery ligation models. In our present study, we used a transplant arteriosclerosis mouse model, which is a severe vascular injury model mediated by alloimmune response, leading to the loss of most, if not all, mature SMCs within the vessel wall. We demonstrated that ≈5% to 55% (depending on the regions analyzed) of neointimal SMCs in the allograft are derived from c-Kit^+^ cells. These seemingly contradicting results, however, could be due to the vascular disease models used, the extent and severity of vascular injury, that is, completely loss of mature SMCs or not, as well as the vascular regions analyzed. Thus, the relative contribution of certain cell types to neointima formation could be highly dependent on the severity of the vessel injury. Besides, we also observed a minor population of c-Kit^+^ medial SMCs. We cannot exclude that this minor population, if any, may also contribute to neointimal SMCs in our model, as recent studies have indicated that clonal expansion of a few medial SMCs contribute to atherosclerosis.^48–50,52^ Also, we observed a much higher labeling of medial SMCs by tdTomato in the region adjacent to the graft (zone 2) compared to distant region (zone 1). The expanded c-Kit–derived SMCs in this region may be derived from adventitial tdTomato^+^ cells like SPCs, or even preexisting medial tdTomato^+^ SMCs, both of which could be possible contributors to neointimal tdTomato^+^ SMCs.

The origin of c-Kit–derived SMCs was further proved to be recipient mice, but not donor grafts. One possible explanation is that cells from donor grafts including ECs, SMCs, and even SPCs may undergo apoptosis due to severe alloimmune response.^23–26^ During this process, inflamed or apoptotic vascular cells might release cytokines to recruit recipient c-Kit^+^ cells to the injury sites, which is supported by our results that increased levels of cytokines such as SCF and TGF-β1 were detected in both grafts and peripheral blood. Bone marrow is an important source of SPCs, however, it remains controversial whether newly formed neointimal SMCs arise from bone marrow-derived SPCs.^9,23,55–58^ Our data revealed that c-Kit–derived SMCs are not from bone marrow, as bone marrow-derived c-Kit^+^ cells rarely co-stained with SMC markers in neointima, supporting the conclusion that neointimal SMCs are derived from nonbone marrow tissues.^23,56–58^ Although c-Kit–derived SMCs do not arise from bone marrow, we did observe bone marrow-derived c-Kit^+^ cells in neointima, which was further suggested to be CD45^+^ leukocytes that may mediate alloimmune responses and drive neointima formation. Despite the fact that most bone marrow-derived c-Kit^+^ cells give rise to CD45^+^ leukocytes in our chimeric mouse model, a number of tdTomato^−^CD45^+^ cells were also detected (Figure 3C). We speculate that other c-Kit^−^ cells, or even c-Kit^+^ cells from nonbone marrow tissues like the vessel wall, adjacent lymph nodes, or spleen, may also contribute to the total CD45^+^ cells in the graft. Although our in vivo data highlight the importance of recipient c-Kit–derived SMCs, the exact origin of these cells remains unclear. Our results that a significant higher proportion of c-Kit–derived neointimal SMCs in graft regions proximal to carotid artery than distal regions (Figure 2) suggest c-Kit^+^ cells may probably migrate from adjacent carotid artery to the graft. Supporting this notion is the finding by Hagensen et al^59^ that flanking recipient vasculature contributes to smooth muscle accumulation in murine allograft vasculopathy. Alternatively, these c-Kit–derived SMCs may also be derived from perivascular tissues, including adventitia and perivascular adipose tissues, which also contain an abundant population of stem cells (data not shown). The transplanted vessels are embedded with recipient perivascular tissues that may directly migrate into intima, where they differentiate into SMCs. Other possible sources include adipose tissue, spleen, and liver that have been reported to harbor SPCs,^60–62^ among which we have also identified tdTomato-labeled c-Kit^+^ cells in the spleen and liver. Further investigation will be needed to confirm the exact origin and identity of these c-Kit–derived SMCs.

We showed that the proportion of c-Kit–derived neointimal SMCs varies in different regions, suggesting that neointima formed in different regions may be heterogeneous and consist of neointimal SMCs from different lineages. A significant higher proportion of c-Kit–derived SMCs were observed in a more severe and irregular neointimal region (zone 3), indicating a possibility that c-Kit–derived SMCs may be related to more disorganized and severe neointima. We also observed a population of adventitial c-Kit–derived SMCs, which showed a more consistent features across different zones and could be a distinct population from neointimal c-Kit–derived SMCs. Thus, it would very be interesting to further investigate the heterogeneity and clonality of c-Kit^+^ cells, such as using a multicolor lineage tracing model.

The mechanisms underlying c-Kit^+^ cell homing to lesion sites and further differentiation into SMCs were also investigated in our study. Our data showed a significant increase of SCF with infiltration of c-Kit^+^ cells in the lesion sites 4 weeks after transplantation. Moreover, migrating c-Kit^+^ cells were readily observed in SCF-abundant adventitia only one day after transplantation, suggesting that SCF may serve as a chemotactic factor for c-Kit^+^ cells in our allograft model. Our in vitro studies further demonstrated that SCF induces c-Kit^+^ cell migration through SCF/c-Kit signaling. Amelioration of neointima formation in vivo as well as c-Kit^+^ cell migration in vitro by ACK2 treatment highlights the importance of SCF/c-Kit signaling in this process, consistent with some previous studies using wire-injury models.^63,64^ SCF has previously suggested to be mainly expressed by ECs and fibroblasts throughout the body.^17^ Our data showed that SCF is expressed in intimal ECs, a small population of SMCs and adventitial c-Kit^+^ cells under normal and diseases conditions (Online Figure XVI; Figure 5A). Thus, we speculate that recipient c-Kit^+^ cells might be guided by SCF, which may be secreted by inflamed or injured above-mentioned cells from donor graft in response to alloimmunity, and recruited to lesion sites to promote neointima formation. Our in vitro data showed the SCF/c-Kit axis also triggers a possible downstream activation of small GTPases, MEK1/2-ERK1/2-MLC, and JNK/c-Jun signaling pathways to regulate cytoskeleton rearrangement, myosin contractibility, and MMP-2 secretion, which eventually leads to cell migration (Online Figure XXV).

Despite the effect of SCF on cell migration, SCF does not induce c-Kit^+^ cell differentiation into SMC in vitro. TGF-β1 has been reported to be significantly increased in vascular diseases.^8,32–34^ In our allograft mouse model, similar results were obtained as seen by the accumulation of TGF-β1 in local neointimal lesions and peripheral blood. Our data further demonstrate that TGF-β1 could promote c-Kit^+^ cell differentiation into SMCs, but not cell migration in vitro. Glucose metabolism was reported to be elevated in atherosclerotic lesions as well as neointimal lesions.^65–67^ Moreover, alteration in cellular metabolism has been reported to regulate cell proliferation, differentiation, and function.^35–37^ We observed significant increases in glucose metabolism and glycolysis during TGF-β1–induced c-Kit^+^ cell differentiation into SMC. Importantly, both glucose metabolism and SMC differentiation could be effectively abrogated by 2-DG or HK-1 knockdown, suggesting a critical role of HK-1–dependent glucose metabolism in TGF-β1–induced cell differentiation (Online Figure XXVB).

Increasing glucose metabolism in c-Kit^+^ cell may provide biosynthetic substrates as well as energy for cell growth and differentiation. Moreover, cellular metabolism can also provide substrates for posttranslational modifications of proteins such as acetylation, methylation, and glycosylation.^41^ The hexosamine biosynthetic pathway is a branch from glucose metabolism that produces UDP-GlcNAc as a substrate for protein O-GlcNAcylation.^42^ We showed that O-GlcNAcylation of total proteins was increased in TGF-β1–treated cells and that blocking O-GlcNAcylation by DON reversed TGF-β1–induced cell differentiation, indicating a possible role of O-GlcNAcylation in regulating SMC differentiation. Interestingly, we further identified that SRF and myocardin, both of which are important regulators in SMC differentiation, could be modified by O-GlcNAcylation. Previous reports have indicated possible modification of SRF by O-GlcNAcylation,^68^ but how this modification may affect SRF remains unknown. It has also been reported that both O-GlcNAcylation and phosphorylation can modify serine or threonine residues of proteins, and that they may sometimes exert opposite roles in modulating intracellular signaling and gene transcription.^69^ While phosphorylation of SRF may either promote or inhibit its transcriptional activity, phosphorylation of myocardin was shown to decrease SMC gene expression.^70^ Therefore, we propose that a balance between O-GlcNAcylation and phosphorylation of SRF and myocardin may alter their transcriptional activity to regulate SMC gene expression (Online Figure XXVB). Further investigations are still needed to prove the functional role of O-GlcNAcylation of SRF and myocardin in SMC differentiation. Interestingly, increased HK-1 and O-GlcNAcylation of myocardin were also detected in a population of c-Kit–derived neointimal SMCs compared to adventitial c-Kit^+^ cells, suggesting that these in vitro mechanisms might happen in vivo. Our data here provide a possible mechanism for c-Kit^+^ cell migration and differentiation, however, we cannot exclude that other mechanisms, or multiple mechanisms synergistically, contribute to this process. Also, it should be noted that the metabolic effects of TGF-β1 are not only restricted to c-Kit^+^ cells, as metabolic reprogramming driven by TGF-β1 has also been detected or suggested in other cells involved in vascular remodeling^71–74^ (Online Figure XXII).

Although our current study demonstrates the critical role of c-Kit^+^ cells in transplant arteriosclerosis, our finding that vascular resident c-Kit^+^ cell is a heterogeneous population makes it difficult to interpret which nonbone marrow c-Kit population generates SMCs in our current study. This is a technical limitation of the lineage tracing mouse model we used, as c-Kit did not specifically label vascular SPCs as expected. To demonstrate the role of vascular SPCs in the generation of neointimal SMCs, further genetic lineage tracing with dual recombinase system^75^ could be used to solve this problem.

In summary, we have demonstrated here that recipient nonbone marrow sources of c-Kit^+^ cells give rise to SMCs and contribute to neointima formation in an allograft transplantation model. Activation of the SCF/c-Kit axis promotes c-Kit^+^ cell migration, whereas TGF-β1–driven glucose metabolism induces cell differentiation into SMCs in vitro. These findings may provide novel insights into the pathogenesis of neointima formation and have further potential therapeutic implications for vascular diseases.

## Editor’s Note

After Online First publication of this manuscript, a problem with the Western blot of myocardin (101 kDa) in Figure 8 was brought to the attention of the authors, who requested the panel be removed in the final published manuscript.

## Acknowledgments

Some figures were produced using Servier Medical Art under a Creative Commons Attribution 3.0 Unported License.

## Sources of Funding

This study was supported by grants from British Heart Foundation (RG/14/6/31144), National Natural Science Foundation of China (81220108004, 81570249, 91339102, 91639302, 91539103, and 31830039), and Royal Society-Newton Advanced Fellowship (NA170109).

## Disclosures

None.

## Supplementary Material

## Novelty and Significance

### What Is Known?

- Neointimal lesions of allograft vessels mainly consist of smooth muscle cells (SMCs).
- c-Kit^+^ cells are present in bone marrow and most tissues/organs.
- Whether c-Kit^+^ cells can differentiate into SMCs is unknown.
