# Supplementary material for: Recipient c-Kit Lineage Cells Repopulate Smooth Muscle Cells of Transplant Arteriosclerosis in Mouse Models
Source: Circ Res. 2019 May 13;125(2):223–41. doi: 10.1161/CIRCRESAHA.119.314855 (PMC6615935; doi:10.1161/CIRCRESAHA.119.314855)
Supplement: Supplementary file 1 [file res-125-223-s001.pdf]

SUPPLEMENTAL MATERIAL

Detailed Methods

Mice and animal procedures.

All mice were maintained at 22°C under a 12-hour light and 12-hour dark cycle and fed with chow diet and water ad libitum. Kit-CreER knock-in mice (C57BL/6 background) were kindly provided by Bin Zhou[1], Institute for Nutritional Sciences, Chinese Academy of Sciences, Shanghai, China. Rosa26-tdTomato mice[2] (B6.Cg-Gt(ROSA)26Sor$^{tm9(CAG-tdTomato)Hze}$/J, 007909) were purchased from the Jackson Laboratory, USA. C57BL/6J and BALB/c mice were purchased from Harlan, Blackthorn, Bicester, UK. Kit-CreER; Rosa26-tdTomato mice (C57BL/6 background) were generated by crossing Kit-CreER mice and Rosa26-tdTomato mice as previously reported[1]. To induce tdTomato expression, Kit-CreER; Rosa26-tdTomato mice were intraperitoneally injected with tamoxifen (Sigma, T5648) dissolved in corn oil (20 mg/ml) for five consecutive days (0.1-0.15 mg/g body weight). Both male and female mice were used in our experiments and were randomly allocated to different experimental groups. All animal experiments performed were approved by the UK Home Office (PPL70/8944).

Bone marrow transplantation was performed as previously described[3]. Briefly, bone marrow cells were harvested by collecting and flushing the cavities of femurs and tibias isolated from donor mice. Bone marrow cells were then passed through 40-μm cell strainers (Falcon, 352340) to obtain single cell suspensions and resuspended in serum-free DMEM (ATCC, 30-2002) before transplantation. A lethal dose of whole-body irradiation (900 rads) was administered to recipient mice. Six hours later, irradiated recipient mice received 5×10^6 donor bone marrow cells via tail vein injection to form chimeric mice.

We performed allograft transplantation as described previously[4]. Briefly, aortic segments were harvested from donor mice, washed and suspended with saline solution containing heparin (100 U/mL). Recipient mice were anesthetized with phenobarbital sodium (50 mg/kg, i.p.). The ventral side of the neck was incised in the midline and muscles were resected, followed by immobilization of the right common carotid artery by clipping at both distal and proximal ends. After dissection in the middle of the artery, both distal and proximal ends were placed with artery cuffs. The aortic segments from donor mice were then implanted between two ends of the artery by sleeving over the artery cuff. Aorta allograft transplantation was performed between BALB/c mice, mice with a C57BL/6J background including Kit-CreER; Rosa26-tdTomato mice, Kit-CreER; Rosa26-tdTomato mice pretreated with tamoxifen, chimeric mice described above and wildtype C57BL/6J mice.

For experiments in which mice were treated with ACK2 (Rat anti-mouse c-Kit monoclonal IgG2b kappa antibody, eBioscience, 14-1172-85) and control IgG antibodies (Rat IgG2b kappa Isotype Control, eBioscience, 14-4031-85), aortic segments were first transplanted from BALB/c mice to tdTomato-labeled Kit-CreER; Rosa26-tdTomato mice. After the aortic segments were implanted, 10 μg ACK2 or control IgG in 20% Pluronic F-127 Gel (Sigma, P2443), prepared by mixing 20 μl 0.5 mg/ml AKC2 or control IgG antibody with 20 μl 40% Pluronic F-127 Gel, were delivered to the adventitial side of the transplanted aortic grafts. Blood, bone marrow and tissues including normal aorta, aortic graft with adjacent tissues, carotid artery, lung, spleen and liver were harvested at indicated times for analyses. The investigators harvesting the tissues and collecting the data were blinded to the groups.

Whole mount fluorescence microscopy

Whole mount bright field and fluorescence images of aortas or aortic grafts with adjacent tissues were acquired using a Nikon (SMZ1270) stereo microscope

Immunofluorescence staining.

For immunofluorescence staining of mouse tissues, tissues were first collected, washed with PBS and fixed in 4% paraformaldehyde (PFA, Santa Cruz, sc-281692) at 4°C for 2-3 h. Tissues were then dehydrated in 30% sucrose solution (BDH, 102747E) overnight at 4°C, embedded in

OCT and frozen at -80°C. Tissues were cut at 10-μm thickness using a CryoStar Cryostat (Thermo Scientific). Concerning tissue preparation for *en face* staining, the adventitial layer was carefully harvested from the whole vessel. For the preparation of cell staining, cells were seeded and cultured in 8-well chamber slides (Millipore, PEZGS0816) before harvesting. For staining of migrating cells on transwell filter membrane, transwell filter was first fixed in 4% PFA for 10 min. Transwell filter membrane was then carefully excised and place onto slide for further blocking and staining. Tissues, cells or frozen sections on slides were first fixed in 4% PFA for 10 min, followed by permeabilization and blocking in 5% donkey serum supplemented with 0.1% Triton X-100 for 1 h at room temperature. Tissues, cells or sections were then stained with primary antibodies overnight at 4 °C, then incubated with Alexa Fluor-conjugated secondary antibodies (Invitrogen, 1:500) for 1 h, followed by DAPI (Molecular Probe, D1306) staining for 8 min. All slides were mounted with anti-fade mounting medium (Dako, s3023). Images were acquired by a Leica (TCS SP5) confocal microscope. For quantification of immunostaining images, images from 3-6 different sections were processed and analysed using Image J[5]. For each section, at least 3 random fields of view (387.5x387.5 μm^2 per field) were counted and the mean values were taken as the data point. Primary antibodies used in this study included c-Kit (R&D, AF1356, 1:50), c-Kit (Santa Cruz, sc-5535, 1:50), CD34 (BD Pharmingen, 553731, 1:100), CD34 (Santa Cruz, sc-7045, 1:100), Sca-1 (Abcam, ab51317, 1:200), tdTomato (Rockland, 600-401-379, 1:500), PDGFR-α (CD140a, Invitrogen, 14-1401-82, 1:100), CD45 (Abcam, ab10558, 1:100), α-SMA (Sigma, A5228, 1:100), SM22 (Abcam, ab14106, 1:100), SM22 (Abcam, ab10135, 1:100), Calponin (Abcam, ab46794, 1:100), CD31 (BD Pharmingen, 553370, 1:100), HK1 (Novus Biologicals, NBP1-51644, 1:100), Myocardin (Sigma, SAB4200539, 1:100), O-Linked N-Acetylglucosamine (O-GlcNAc, Abcam, ab2739, 1:1000), SCF (Santa Cruz, sc13126, 1:100), TGFβ1 (Abcam, ab27969, 1:100), Vinculin (Abcam, ab18058, 1:50), p-FAK (Abcam, ab81298, 1:100) and Phalloidin-AF488 (Invitrogen, A12379). Alexa Fluor-conjugated secondary antibodies used in this study included Donkey anti-Mouse IgG (Invitrogen, A-21202 for Alexa Fluor 488, A10036 for Alexa Fluor 546, A-21203 for Alexa Fluor 594, A-31571 for Alexa Fluor 647), Donkey anti-Goat IgG (Invitrogen, A-11055 for Alexa Fluor 488, A-11056 for Alexa Fluor 546, A-11058 for Alexa Fluor 594, A-21447 for Alexa Fluor 647), Donkey anti-Rabbit IgG (Invitrogen, A-21206 for Alexa Fluor 488, A10040 for Alexa Fluor 546, A-21207 for Alexa Fluor 594, A-31573 for Alexa Fluor 647), Donkey anti-Rat IgG (Invitrogen, A-21208 for Alexa Fluor 488, A-21209 for Alexa Fluor 594). A secondary antibody only control was used for all immunostaining.

Flow cytometry analysis.

Isolation of cells from the aorta or aortic graft was performed by cutting aorta into 1-mm pieces, followed by collagenase I (Gibco,17018029) digestion at 37°C for 30 min. During this process, frequent stirring of artery pieces was performed to ensure full digestion of tissues. Bone marrow cells were isolated via thorough flushing of PBS through mouse femurs and tibias. Cells were then passed through a 40-μm cell strainer and incubated with red blood cell lysis buffer (eBioscience, 00-4333) to remove red blood cells. All prepared cell samples were kept on ice before flow cytometric analysis. For flow cytometric staining, cells were stained with conjugated-antibodies at 1:50 dilution for 30 min on ice. Antibodies used in this study include α-SMA-AF488 (Abcam, ab184675), c-Kit-PE (Biolegend, 105807), Sca-1-PE (BD biosciences, 553108), CD29-FITC (Abcam, ab21845), CD34-FITC (BD biosciences, 560238), CD105-PE (Biolegend, 120407), CD140a-PE (eBioscience, 12-1401-81), CD146-FITC (Biolegend, 134705), CD201-PE (eBioscience, 12-2012-80), CD45-AF647 (Biolegend, 103124), Ki-67-FITC (eBioscience, 11-5698-82). Corresponding isotype antibodies including FITC Rat IgG2a, κ (BD biosciences, 555843), FITC Mouse IgG1, κ (Biolegend, 400108), PE Rat IgG2a, κ (eBioscience, 12-4321-41), PE Rat IgG2b, κ (Biolegend, 400607), AF647 Rat IgG2b, κ (Biolegend, 400626) and AF488 Mouse IgG2a (Abcam, ab237608) were used as control. After centrifugation and supernatant removal, cells were suspended in PBS and analyzed with a BD LSR Fortessa II flow cytometer (Becton Dickinson). To measure glucose uptake or mitochondrial mass, 2-NBDG (100 μM, Invitrogen, N13195) or MitoTracker Green FM (20

nM, Invitrogen, M7514) was directly added to cells and incubated in 5% CO2 incubator at 37°C for 1 h. Cells were then detached by trypsin, washed with PBS and analyzed by BD ACCURI C6 flow cytometer. All flow cytometry data were then analyzed using FlowJo software (Tree Star).

Mouse vascular c-Kit^{+} cell isolation, cell culture and differentiation.

Isolation of mouse vascular c-Kit^{+} cells were performed as previously described[6]. Briefly, vessel grafts from recipient mice were harvested, cut into pieces and flattened on a T25 flask coated with 0.04% gelatin (Sigma, G1393) and maintained in complete stem cell culture medium, which consists of DMEM (ATCC, 30-2002), 10% EmbryoMax ES Cell Qualified FBS (Millipore, ES-009-B), 10 ng/mL leukemia inhibitory factor (Merck Millipore, LIF1050), 0.1 mM 2-mercaptoethanol (GIBCO, 31350-010), 100 U/mL penicillin-streptomycin (GIBCO, 15140122) and 2 mM L-glutamine (GIBCO, 25030081). Explanted vessel grafts were maintained in T25 flask for around one week for the outgrowth of cells before the first passage. Cells derived from the outgrowth of graft tissues were then sorted with c-Kit (Miltenyi Biotec, 130-091-224) microbeads according to manufacturer's instructions. Sorted c-Kit^{+} cells were then cultured in complete stem cell culture medium and passaged at a ratio of 1:3 every 3 days. Cell culture medium was changed every other day. The passage numbers of c-Kit^{+} cells we used in cell experiments were between passages 5 to 15. Both wildtype and tdTomato-labeled c-Kit^{+} cells were isolated. For cell differentiation experiments, c-Kit^{+} cells were cultured on gelatin-coated plates and maintained in complete differentiation medium, which consists of DMEM (GIBCO, 31966-021), 10% FBS (GIBCO, 10270), 0.2 mM 2-mercaptoethanol, 100 U/mL penicillin-streptomycin, in the presence of 2 ng/ml recombinant mouse TGFβ1 (R&D systems, 7666-MB-005) or 100 ng/ml (or indicated doses) of mouse stem cell factor (SCF, Peprotech, AF-250-03). Cells were treated with indicated doses of inhibitors including ACK2 antibody (1 μg/ml), control IgG antibody (Rat IgG2b kappa isotype control, 1 μg/ml), 2-Deoxy-D-glucose (2DG, Sigma, D8375) and CP-91149 (Sigma, PZ0104) where indicated.

Mouse aortic SMC and peritoneal macrophage isolation and cell culture

Isolation of mouse aortic SMC was performed as previously described[7]. Briefly, aorta was harvested from 8-week-old C57BL/6J mice. After the adventitial and intimal layers were carefully removed, the remaining media was cut into pieces, explanted on a 0.04% gelatin-coated T25 flask and maintained in DMEM (GIBCO, 31966-021) with 10% FBS. Explanted tissues were maintained in T25 flask for around one week for the outgrowth of SMCs before the first passage. SMCs were passaged every 2 days. The passage numbers of SMCs used in this study were between passages 3 to 5. Mouse peritoneal macrophages were isolated from the peritoneal cavity of 8-week-old C57BL/6J mice by flushing the peritoneum with PBS with 10% FBS. Peritoneal cells were maintained in DMEM with 10% FBS and seeded in a 6-well-plate and incubated in 5% CO2 incubator at 37°C for for 2-3 h. Nonadherent cells were then removed by gently washing with PBS for three times. To test the effect of TGFβ1 on SMC and macrophage, cells were treated with 2 ng/ml recombinant mouse TGFβ1 for 3 days or indicated times.

Transwell migration assay.

Transwell migration assays were performed using transwell cell culture plates (Corning Costar, 3464) with 8.0-μm pore membrane filters. Cells (1×10^{6} cells/100 μl serum-free medium) were seeded in the upper chamber, while indicated doses of SCF or TGFβ1 in 600 μl serum free medium were then added to the lower chamber. For experiments of c-Kit inhibition, ACK2 or control IgG antibody was added to both upper and lower chambers. After 18-hour incubation, medium from both chambers was discarded. Non-migrating cells remaining on the top of the transwell filters were removed by cotton swabs. The migrating cells on the lower surface of the transwell filter were fixed in 4% PFA for 10 min and then stained with 1% crystal violet (Sigma, HT90132) for 15 min. Images were acquired using Nikon Eclipse TS100 microscope. Cells were counted in 5 random fields under the microscope.

Scratch-wound healing assay.

Cells (1×10^5 cells per well) were seeded in a 12-well plate and maintained in complete medium. Once the cells reached 80% confluence, a wound was made by scratching the well from top to bottom using a 1 mL pipette tip. Images of the scratch wound were obtained under a microscope in 5 random fields. Serum-free medium containing indicated doses of SCF was then added to the cells. After 18-hour incubation, images of the scratch wound were obtained again in 5 random fields. Data shown are the relative mean number of cells migrating into the scratch-wound area at 18-hour time points compared to the initial scratch wound.

Western blot analysis.

Cells were lysed in RIPA buffer (Thermo Fisher, 89901) supplemented with phosphatase inhibitor tablets (Roche, 04906837001) and protease inhibitor tablet (Roche, 05892970001). For extraction of cytoplasmic and nuclear proteins, a Nuclear Extraction Kit (Abcam, ab113474) was used according to manufacturer's instruction. Quantification of protein concentration was performed by DC protein assay kit (Biorad, 5000112) according to manufacturer's instruction. Samples were separated in NuPAGE 4-12% Bis Tris-gels (Thermo Fisher, NP0321) and transferred to nitrocellulose membranes (GE healthcare, RPN303LFP). After blocking in 5% milk or BSA for 1 h, membranes were incubated with primary antibody overnight and treated with appropriate HRP-conjugated or Dye secondary antibody for 1 h at room temperature. Membrane detection was performed either using ECL western blotting detection reagents (GE Healthcare, RPN2106) when incubated with HRP-conjugated (Dako) or IRDye (LI-COR) secondary antibodies, or Odyssey CLx near-infrared fluorescence imaging system when incubated with IRDye secondary antibodies. Primary antibodies used in this study are listed as follows: p-c-Kit (Cell Signaling, 3391S, 1:200), c-Kit (Santa Cruz, sc-5535, 1:100), p-MEK1/2 (Cell Signaling, 9154S, 1:500), MEK1/2 (Cell Signaling, 4694S, 1:1000), p-ERK1/2 (Santa Cruz, sc-16982-R, 1:500), ERK1/2 (Cell Signaling, 4695P, 1:1000), p-JNK (Santa Cruz, sc-6254, 1:1000), anti-JNK (Santa Cruz, sc-474, 1: 1000), p-c-Jun (Santa Cruz, sc-16312, 1:100), c-Jun (Santa Cruz, sc-74543, 1:100), p-MLC (Cell Signaling, 3675S, 1:200), MLC (Cell Signaling, 3672S, 1:500), α-SMA (Sigma, A5228, 1:1000), Calponin (Abcam, ab46794, 1:1000), SM22 (Abcam, ab14106, 1:1000), HK1 (Cell Signaling, 2024S, 1:1000), HK2 (Cell Signaling, 2867S, 1:1000), O-Linked N-Acetylglucosamine (Abcam, ab2739, 1:1000), Myocardin (Abcam, ab22073, 1:1000), SRF (Santa Cruz, sc-335, 1:1000), Lamin B1 (Abcam, ab133741, 1:1000) and GAPDH (Abcam, ab8245, 1:1000).

Co-immunoprecipitation (Co-IP)

Cells were lysed in Pierce IP lysis buffer (Thermo Scientific, 87788) supplemented with protease inhibitor cocktail (Thermo Scientific, 78425) and incubated with Myocardin (Santa Cruz, sc-33766), SRF (Santa Cruz, sc-335), control normal mouse (Santa Cruz, sc-2025) or rabbit (Santa Cruz, sc-2027) IgG antibodies before immunoprecipitation with Protein A/G PLUS-Agarose (Santa Cruz, sc-2003). Immunoprecipitates were then washed with IP lysis buffer for five times, resuspended in SDS sample buffer, and resolved by western blot as described above.

G-LISA RhoA/Rac1/Cdc42 activation assay.

The activation of GTPase family was measured by G-LISA activation assay kit (Cytoskeleton) including RhoA (BK124-S)/Rac1 (BK128-S)/Cdc42 (BK127-S) according to manufacturer's instruction. Briefly, cell lysate was collected and protein was extracted as described above. Protein concentration was measured by detection solution provided in the kit. Equalized lysates, buffer blank and positive control were then added to a 96-well plate. The plate was placed on an orbital shaker at 400 rpm for 45 min at 4 ºC. Samples were then mixed with antigen presenting buffer for 2 minutes and incubated with diluted anti-RhoA/Rac1/Cdc42 primary antibody on an orbital shaker at 400 rpm for 45 min. Samples were then incubated with

secondary antibody for 45 min, and HRP detection reagent for 15 min. All reactions were terminated by adding HRP Stop Buffer. Measurement of absorbance was read at 490 nm.

Mouse serum ELISA assay.

Blood plasma concentrations of SCF and TGFβ1 were measured via Mouse SCF Quantikine ELISA Kit (R&D, MCK00) and Mouse TGFβ1 ELISA Kit (Abcam, ab119557). Levels of MMP-2 and MMP-9 from cell culture supernatant were detected by Total MMP-2 Quantikine ELISA Kit (R&D, MMP200) and Mouse Total MMP-9 Quantikine ELISA kit (R&D, MMPT90). BrdU assay was performed using a cell proliferation ELISA kit (Roche, 11647229001). All experiments were performed according to the standard protocols provided by manufacturers.

Conventional polymerase chain reaction.

Genotyping of Kit-CreER; Rosa26- tdTomato mice was performed via conventional polymerase chain reaction (PCR) using the following primers: *Kit-CreER* (forward: 5'-GCCTTCTATCGCCTTCTTGACG-3'; reverse: 5'-CAGTCGGCACAAAAGCATCAC-3'); *Rosa26-tdTomato* (wild type forward: 5'-AAGGGAGCTGCAGTGGAGTA-3'; wild type reverse: 5'-CCGAAAATCTGTGGGAAGTC-3'; mutant forward: 5'-CTGTTCCTGTACGGCATGG-3'; mutant reverse: 5'-GGCATTAAAGCAGCGTATCC-3'.). Amplification of target genes was achieved by a mixture of genomic DNA with specific primers and Taq DNA polymerase. PCR products were then separated on 2% agarose gel (Invitrogen, 16500-500) and observation of target bands was acquired by BioSpectrum Imaging system.

Quantitative Real-Time Polymerase Chain Reaction.

RNA was extracted via a RNeasy Mini Kit (Qiagen, 74106) and then reverse transcribed to cDNA and amplified using a QuantiTect Reverse Transcription Kit (Qiagen, 205311). The procedure of quantitative real-time polymerase chain reaction (qPCR) was performed according to SYBR green system (PCR Biosystems, PB20.16-51). Primer sequences used in this study are as follow: *Tagln* (forward: 5'-GATATGGCAGCAGTGCAGAG-3' and reverse: 5'-AGTTGGCTGTCTGTGAAGTC-3'); *Cnn1* (forward: 5'-GGTCCTGCCTACGGCTTGTC-3' and reverse: 5'-TCGCAAAGAATGATCCCGTC-3'); *Acta2* (forward: 5'-TCAGGGAGTAATGGTTGGAATG-3' and reverse: 5'-GGTGATGATGCCGTGTTCTA-3'); *Myh11* (forward: 5'-CGGCAACTGGTATCCAATCT -3' and reverse: 5'-GTCTCTCTCATCCGCATACTTG-3'); *Kit* (forward: 5'-CCTCCTGCCCTTTATCCTTTAG-3' and reverse: 5'-GACCTCCAAACCAGCTTACTT-3'); *Ly6a* (forward: 5'-AGGAGGCAGCAGTTATTGTGG-3' and reverse: 5'-CGTTGACCTTAGTACCCAGGA-3'); *Cd34* (forward: 5'-CACAACCACAGACTTCCCCA-3'and reverse: 5'-CCTTAATGGCACTCGGAGCA-3'); *Hk1* (forward: 5'-ACCAACCCACAAAACAACGC-3' and reverse: 5'-CCCAAGGAAACACCACTCCG-3'); *Hk2* (forward: 5'-ATGATCGCCTGCTTATTCACG-3' and reverse: 5'-CGCCTAGAAATCTCCAGAAGGG-3'); *Hk3* (forward: 5'-GATTTCGGTTAAGTGGCTACAGAG-3' and reverse: 5'-ATTGCTGCAAGCATTCCTGGG-3'); *Hk4* (forward: 5'-AGTGCAGAAGTCCTTGGCTG-3' and reverse: 5'-CTGAGGATAAGCAGGGGTCG-3'); *Pygb* (forward: 5'-TATGCGGGTGGAAGATGTCG-3' and reverse: 5'-CGTCCTTGAAGCAGTCTGGA-3'); *Pygl* (forward: 5'-GAGTAGAGCACACCCAGACG-3' and reverse: 5'-CCAGAGGCGCATAGTGT-3'); *Pygm* (forward: 5'-AGGATCGCAATGTGGCTACT-3' and reverse: 5'-CCTTTTCGTAGTAATGCTGCTGT-3'); *Gapdh* (forward: 5'-TCTCCCTCACAATTTCCATCC-3' and reverse: 5'- GGGTGCAGCGAACTTTATTG-3').

Extracellular flux analysis.

Oxygen consumption rate (OCR) and extracellular acidification rate (ECAR) were analyzed using a Seahorse XFe24 Analyzer (Seahorse Bioscience) as previously described. Cells were seeded in a gelatin-coated XF24 cell culture microplate and treated with the indicated reagents

for the indicated times. For real-time monitoring of metabolic changes upon TGFβ1 treatment, tests were performed under basal conditions and in response to 2 ng/ml TGFβ1. Mitochondrial stress tests were performed under basal conditions and in response to 1 μM oligomycin (Sigma, 75351), 1 μM carbonyl cyanide 4-trifluoromethoxy phenylhydrazone (FCCP, Sigma, C2920), 1 μM rotenone (Sigma, R8875), and 1 μM antimycin (Sigma, A8674). Glycolysis stress tests were performed under basal conditions and in response to 10 mM glucose (Sigma, G7021), 1 mM oligomycin, and 100 mM 2DG. Cell numbers were counted after metabolic assay and used for data normalization. Wave software (Seahorse Bioscience) was used to analyze OCR and ECAR in all metabolic stress tests. For analysis of ECAR in mitochondrial stress tests, basal ECAR was the basal rate before injection of metabolic reagents, and maximal ECAR was calculated after the injection of rotenone and antimycin A.

siRNA transfection.

Pre-designed siRNAs including HK1 (4390771 Assay ID s67555), HK2 (4390771 Assay ID s67558), KIT (4390771 Assay ID s68808) and Silencer™ Select Negative Control No. 1 (4390843) siRNA were all purchased from ThermoFisher Scientific. Cells were transfected with siRNA using a Lipofectamine RNAiMAX reagent (Invitrogen, 13778150) in Opti-MEM™ I reduced serum medium (GIBCO, 31985070) according to the manufacturer's instructions. After 5-hour incubation at 37°C, the medium was changed to complete differentiation medium and harvested 2 days after transfection for further analysis.

Hexokinase activity.

Hexokinase activity was measured using a Hexokinase Activity Assay Kit (Abcam, ab136957) according to the manufacturer's instructions. In brief, cells were harvested and homogenized in assay buffer. Protein concentrations of the cell lysates were then determined and 40 μg protein was mixed with the reaction mixture in a 96-well microplate. The increase in absorbance by NADH at 450 nm was measured and calculated to determine pyruvate kinase activity.

Statistical analyses

All data are shown as mean ± SEM. Statistical analysis was performed using GraphPad Prism 7 (GraphPad Software). All data were first analyzed for normality by Shapiro-Wilk test ($3 \leq n < 8$) or D'Agostino & Pearson test ($n \geq 8$), followed by unpaired student's t test for comparison between two groups, and one-way ANOVA with Dunnett's or Tukey's post hoc tests, or two-way ANOVA with Bonferroni's test for comparison among multiple groups, as indicated in the figure legend. $P<0.05$ was considered to be statistically significant.

Supplemental References

1. Liu Q, Huang X, Zhang H, Tian X, He L, Yang R, Yan Y, Wang QD, Gillich A, Zhou B. C-kit(+) cells adopt vascular endothelial but not epithelial cell fates during lung maintenance and repair. *Nat Med*. 2015;21:866-868
2. Madisen L, Zwingman TA, Sunkin SM, Oh SW, Zariwala HA, Gu H, Ng LL, Palmiter RD, Hawrylycz MJ, Jones AR, Lein ES, Zeng H. A robust and high-throughput cre reporting and characterization system for the whole mouse brain. *Nat Neurosci*. 2010;13:133-140
3. Hu Y, Davison F, Ludewig B, Erdel M, Mayr M, Url M, Dietrich H, Xu Q. Smooth muscle cells in transplant atherosclerotic lesions are originated from recipients, but not bone marrow progenitor cells. *Circulation*. 2002;106:1834-1839
4. Dietrich H, Hu Y, Zou Y, Dirnhofer S, Kleindienst R, Wick G, Xu Q. Mouse model of transplant arteriosclerosis: Role of intercellular adhesion molecule-1. *Arterioscler Thromb Vasc Biol*. 2000;20:343-352.
5. Rueden CT, Schindelin J, Hiner MC, DeZonia BE, Walter AE, Arena ET, Eliceiri KW. Imagej2: Imagej for the next generation of scientific image data. *BMC Bioinformatics*. 2017;18:529
6. Hu Y, Zhang Z, Torsney E, Afzal AR, Davison F, Metzler B, Xu Q. Abundant progenitor cells in the adventitia contribute to atherosclerosis of vein grafts in apoe-deficient mice. *J Clin Invest*. 2004;113:1258-1265
7. Hu Y, Zou Y, Dietrich H, Wick G, Xu Q. Inhibition of neointima hyperplasia of mouse vein grafts by locally applied suramin. *Circulation*. 1999;100:861-868

Supplemental Figures and Figure Legends

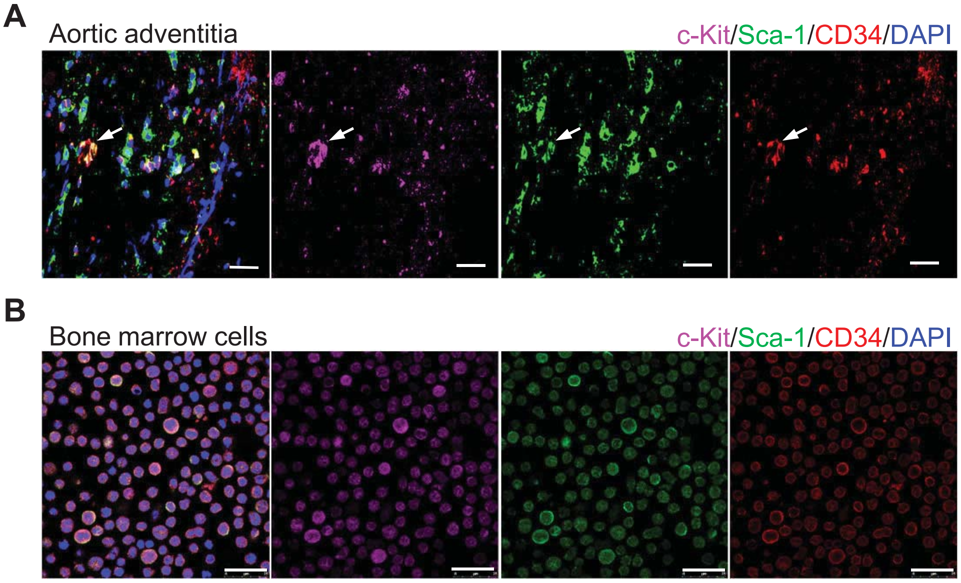

Online Figure I. Staining of c-Kit⁺/Sca-1⁺/CD34⁺ cells in aortic adventitia and bone marrow. (**A**) En face staining of aortic adventitial layer showing cells stained with c-Kit, Sca-1 and CD34 from wildtype C57BL/6J mice (n=3). Scale bars, 50 μm. (**B**) Immunostaining of bone marrow cells from wildtype C57BL/6J mice showing cells stained with c-Kit, Sca-1 and CD34 (n=3). Scale bars, 25 μm.

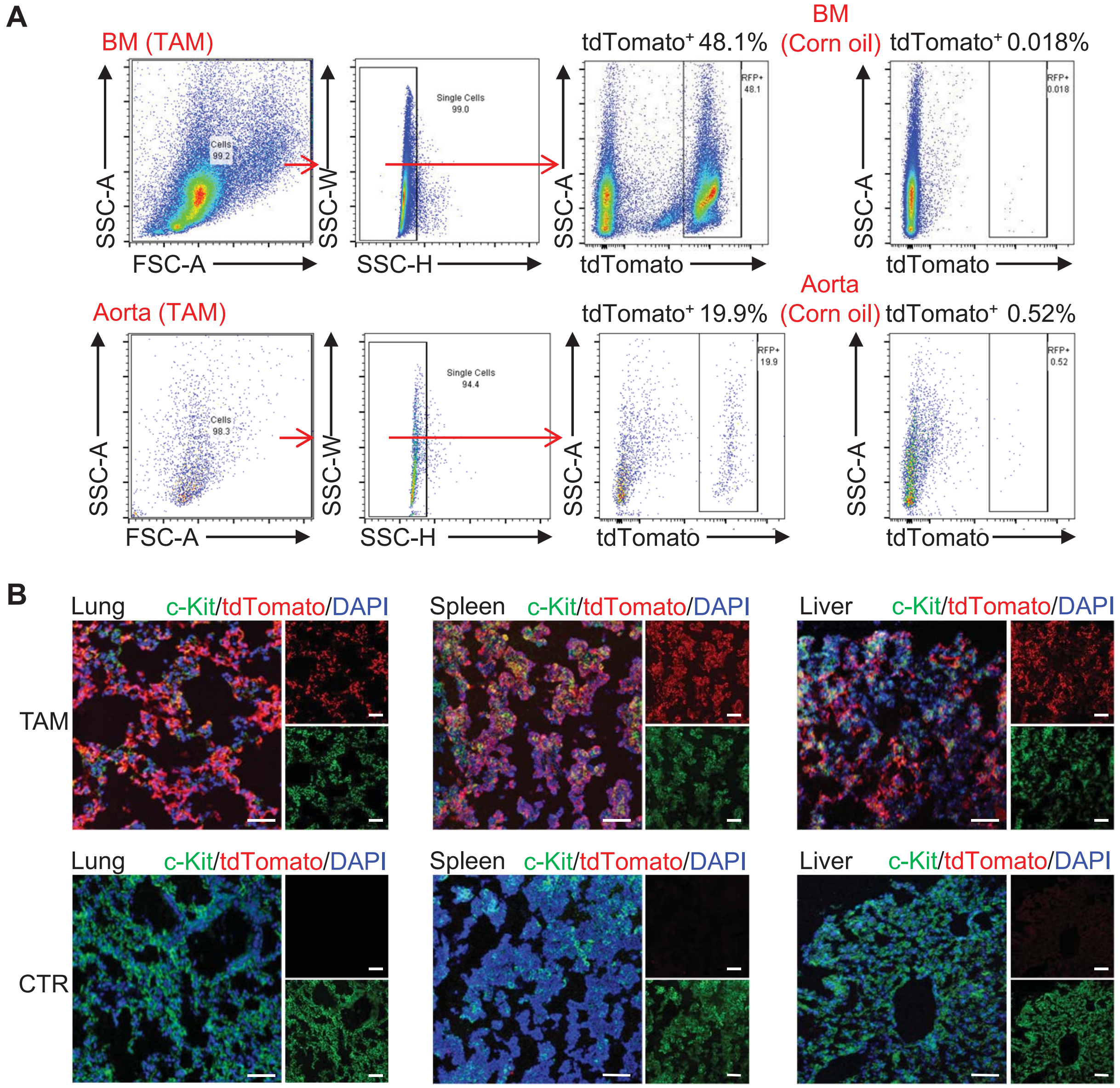

Online Figure II. Verification of tamoxifen-induced tdTomato labeling in Kit-CreER; Rosa26-tdTomato mice. Kit-CreER; Rosa26-tdTomato mice were treated with five pulses of tamoxifen or corn oil and tissues were harvested for analysis as described in Figure 1B. (**A**) Representative gating strategy for flow cytometric analysis of tdTomato$^+$ cells from bone marrow and aortic tissues (n=3). (**B**) Representative images showing tdTomato$^+$ labelling of c-Kit$^+$ cells in lung, spleen and liver from Kit-CreER; Rosa26-tdTomato mice (n=6 per group). Scale bars, 50 µm. CTR indicates corn oil control; TAM, tamoxifen.

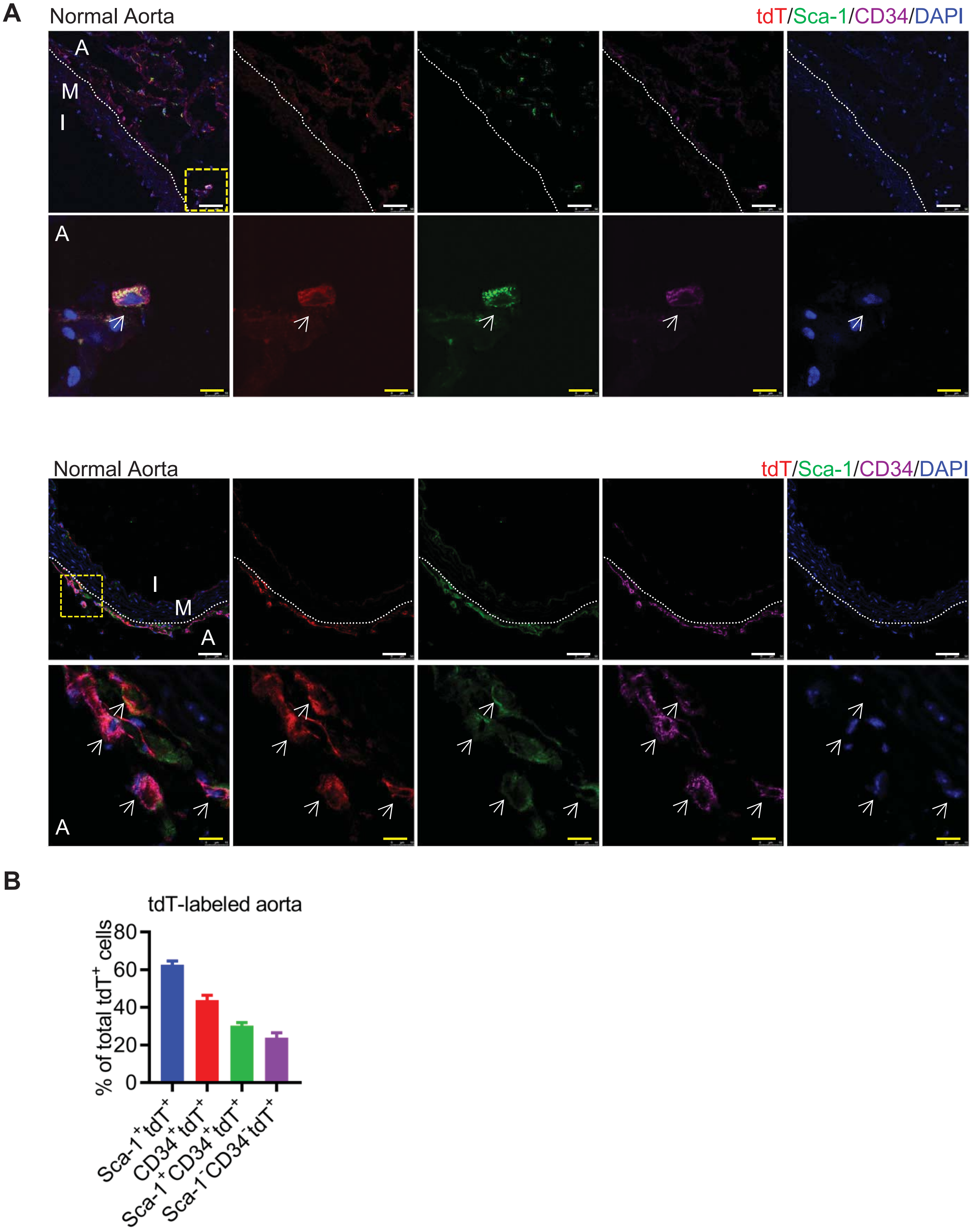

Online Figure III. Related to Figure 1D. Distribution of tdTomato$^+$, Sca-1$^+$ and/or CD34$^+$ cells in the normal aorta. Kit-CreER; Rosa26-tdTomato mice were treated with five pulses of tamoxifen and aortas were harvested for analysis as described in Figure 1B (n=6 per group). (**A**) Representative images showing staining of tdTomato, Sca-1 and CD34 in the aorta. Arrows indicate examples of triple positive cells. Scale bars, 50 μM (white) and 10 μM (yellow). (**B**) Quantification of co-staining cells. Sca-1$^+$tdT$^+$ cells include both Sca-1$^+$tdT$^+$CD34$^+$ and Sca-1$^+$tdT$^+$CD34$^-$ cells, while CD34$^+$tdT$^+$ cells include both CD34$^+$tdT$^+$Sca-1$^+$ and CD34$^+$tdT$^+$Sca-1$^+$ cells. Data represent mean and SEM, n=3. A indicates adventitia; M, media; I, intima; tdT, tdTomato.

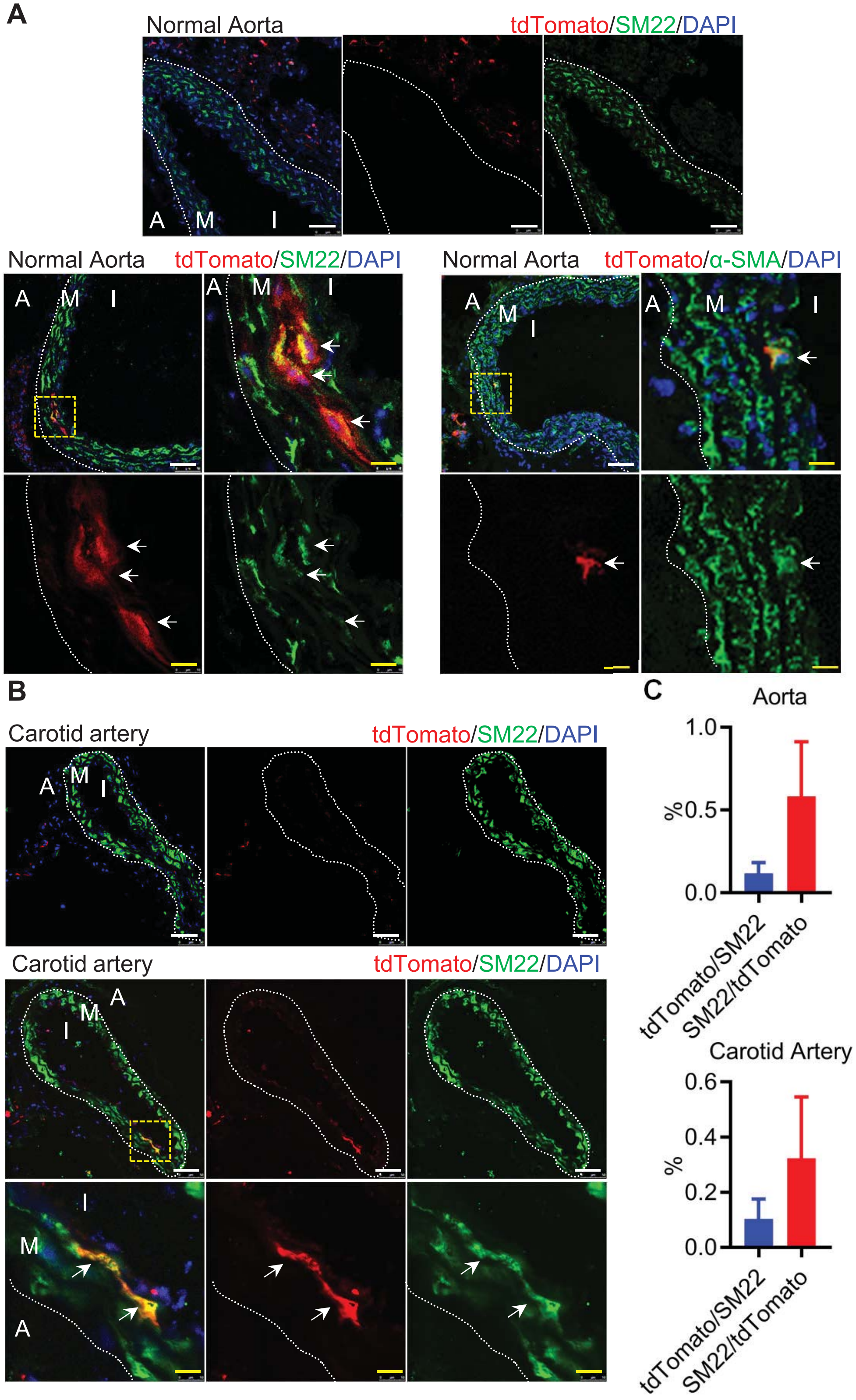

Online Figure IV. Few medial SMCs are tdTomato⁺ cells in the blood vessels under physiological conditions. Kit-CreER; Rosa26-tdTomato mice were treated with five pulses of tamoxifen and tissues were harvested for analysis as described in Figure 1B (n=6 mice per group). (**A**) Representative images showing staining of tdTomato, SM22 and α-SMA in aorta. (**B**) Representative images showing staining of tdTomato and SM22 in carotid artery. (**C**) Quantification of co-staining cells in normal aorta and carotid artery. Data represent mean ± SEM, n=20. Arrows indicate double positive cells. Scale bars, 50 μm (white), 10 μm (yellow). A indicates adventitia; M, media; I, intima or neointima; tdT, tdTomato.

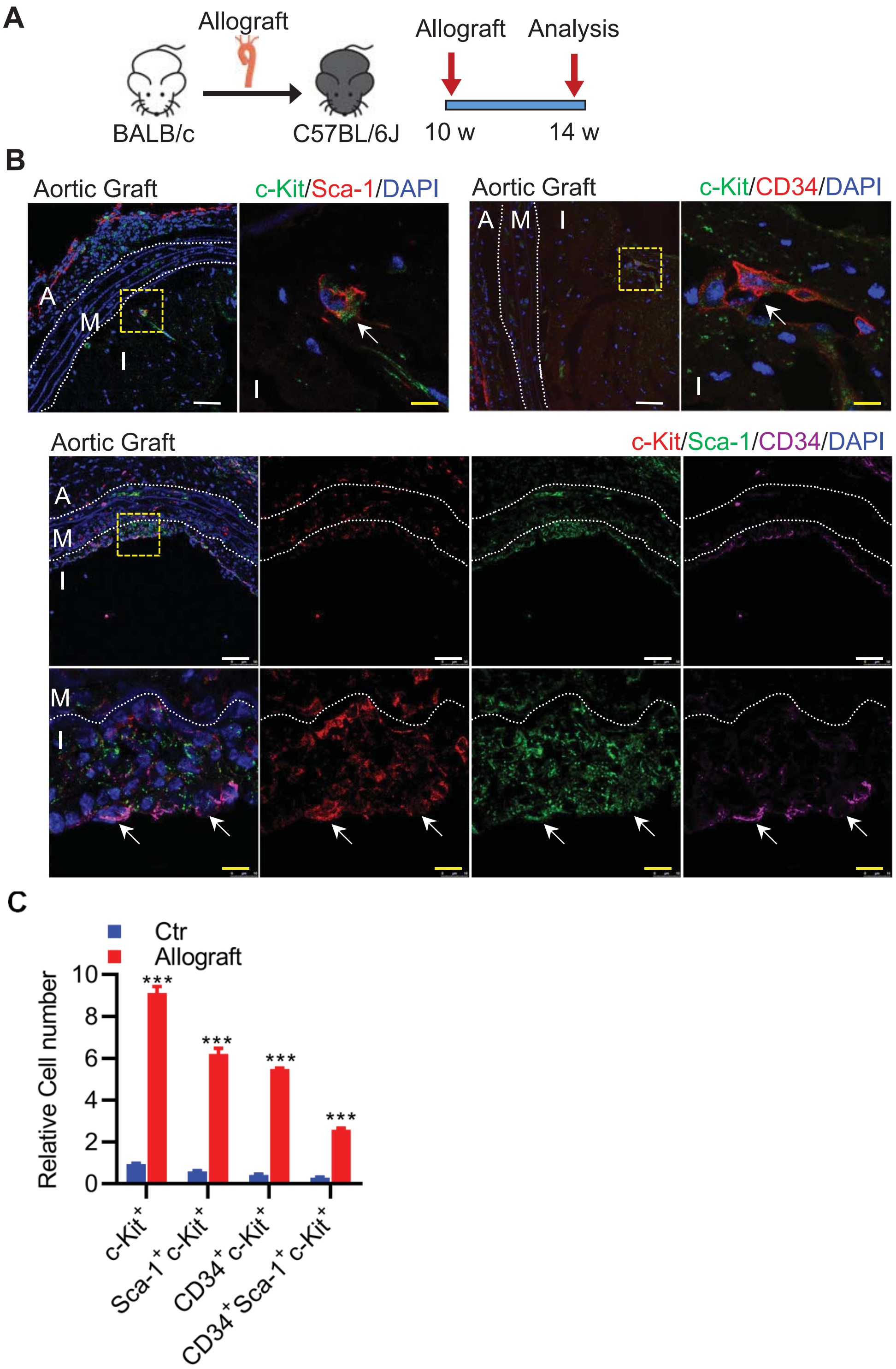

Online Figure V. c-Kit⁺ cells stained with Sca-1 and/or CD34 in transplant arteriosclerosis. (**A**) Schematic showing procedure for allograft transplantation experiments. Aortic segments from BALB/c mice were transplanted into C57BL/6J mice and grafts were collected for analyses 4 weeks after surgery (n=6 per group). (**B**) Representative images of aortic allografts stained for c-Kit, Sca-1 and CD34. Arrows indicate double or triple positive cells. (**C**) Quantification of relative cell numbers in aortic allografts compared to normal aorta. Data represent mean ± SEM. ****P*<0.001, by unpaired two-tailed t test, n= 3 per group. Scale bars, 50 μm (white), 10 μm (yellow). A indicates adventitia; M, media; I, neointima.

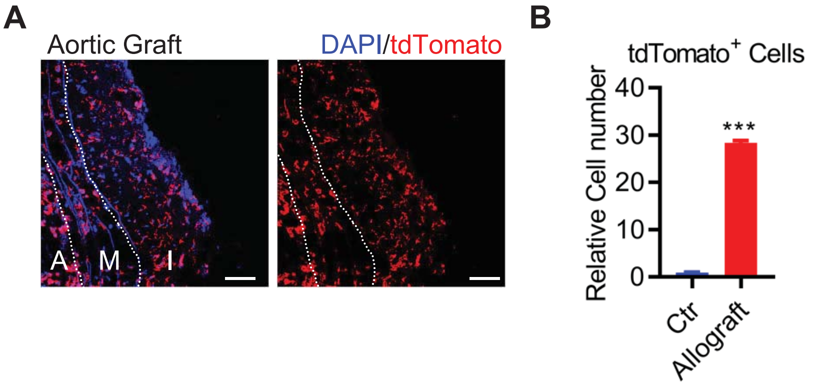

Online Figure VI. Accumulation of tdTomato⁺ cells in aortic allograft. (**A**) Representative images showing tdTomato⁺ cells in the aortic grafts. Scale bars, 50 μm. (**B**) Graph showing quantification of relative cell number of tdTomato⁺ cells in aortic grafts compared to normal aorta. Data represent mean ± SEM. ***P<0.001, by unpaired two-tailed t test, n=12. A indicates adventitia; M, media; **I**, neointima.

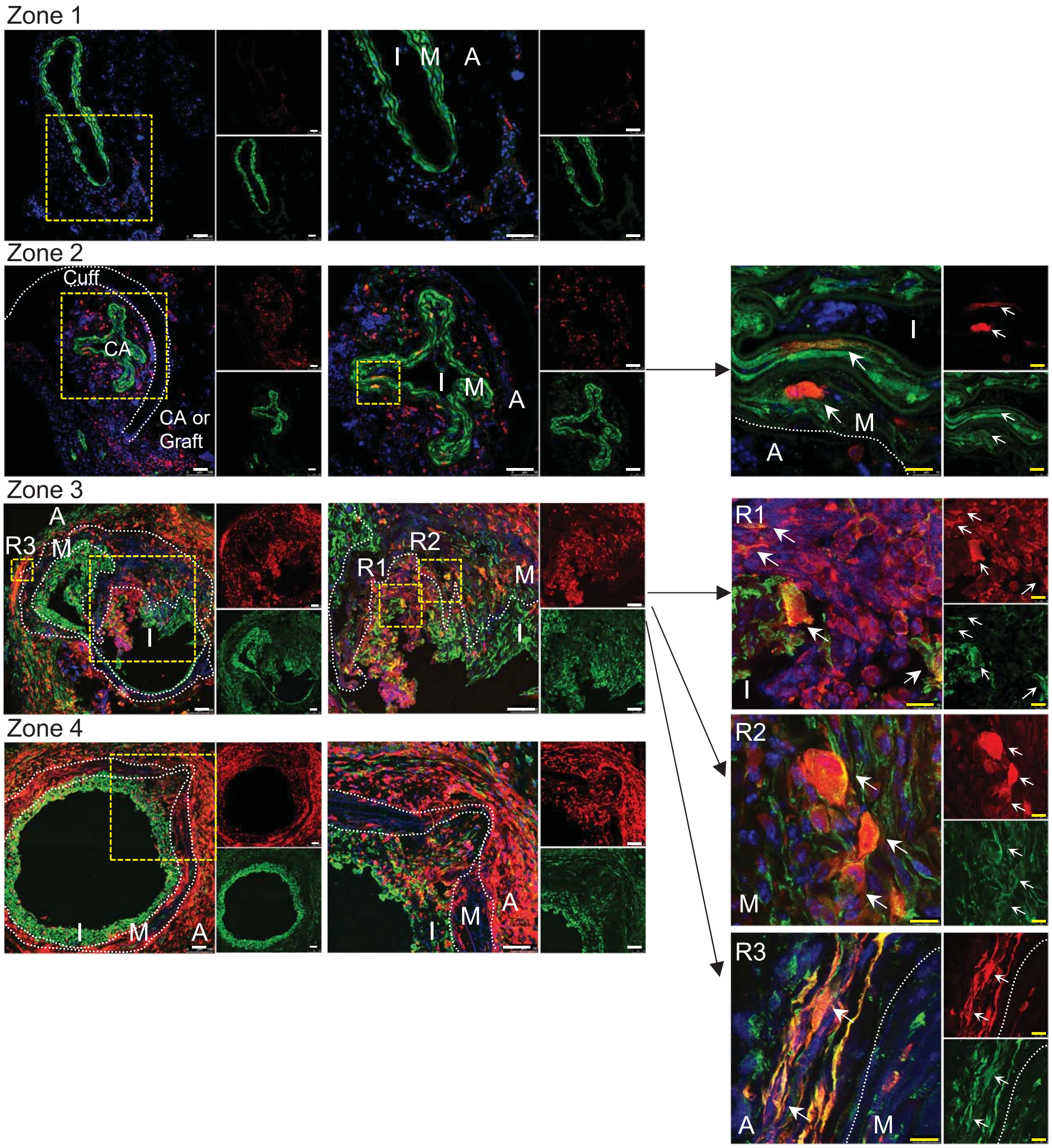

Online Figure VII. Related to Figure 2. Representative images showing c-Kit-derived SMCs in aortic allograft (with lower percentage of tdTomato-labelled neointimal SMCs). Representative images showing different zones of aortic allograft with adjacent carotid arteries, stained with tdTomato and SM22. Compared to Figure 2, these images show similar staining of SM22 and tdTomato in zone 1 and 2, but significant lower tdTomato-labelled neointimal SMCs in zone 3 and 4. Arrows indicate tdTomato^{+}SM22^{+} cells. Scale bars, 50 μm (white) and 10 μm (yellow). A indicates adventitia; M, media; I, intima or neointima; CA, carotid artery; R1-3, region 1-3; tdT, tdTomato.

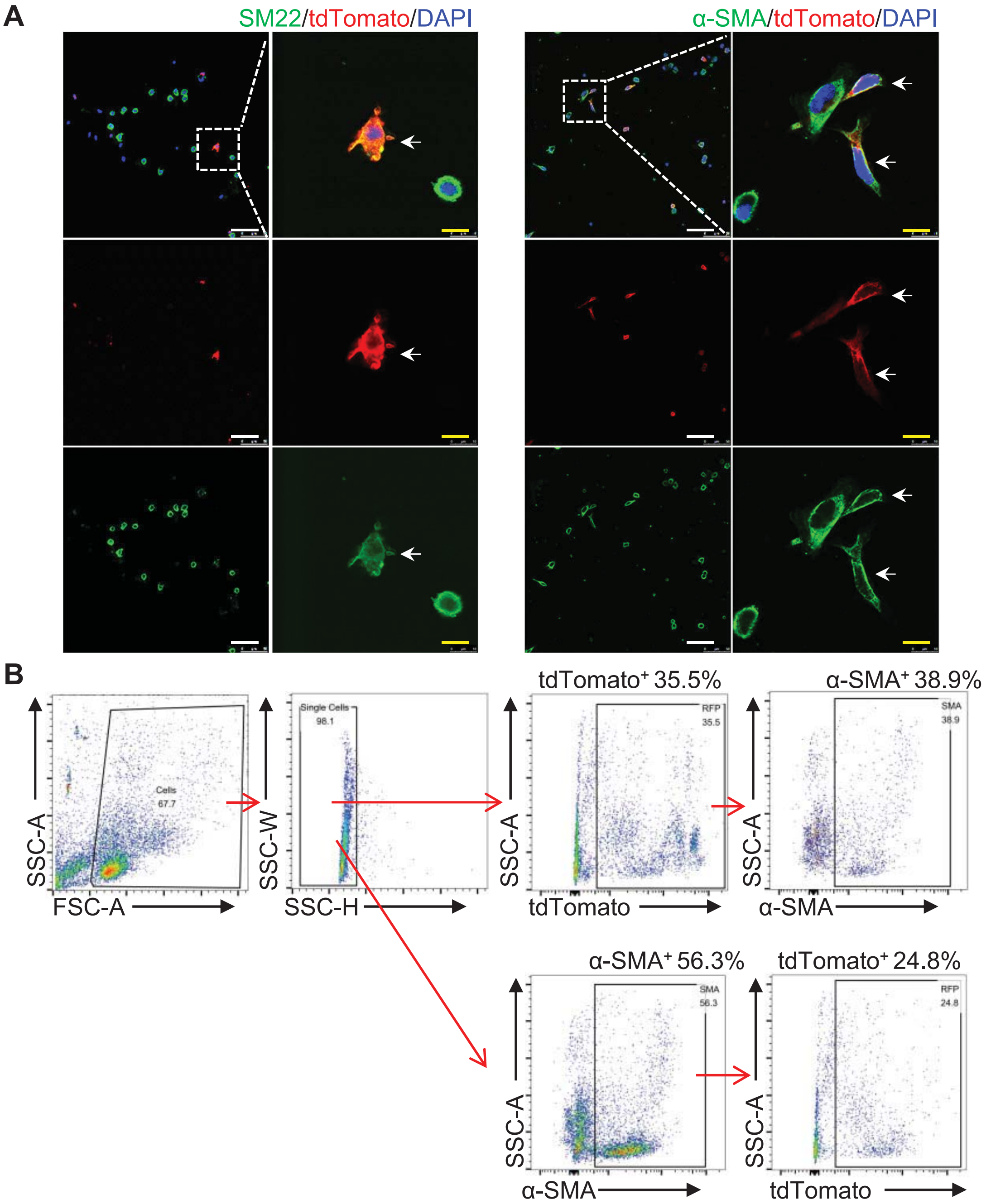

Online Figure VIII. Related to Figure 2. Detection of c-Kit-derived SMCs in aortic allograft. Single cell suspensions from aortic grafts were isolated from Kit-CreER; Rosa26-tdTomato mice described in Figure 2A. (**A**) Representative immunostaining images showing single cells from aortic grafts (n=4) stained with tdTomato, SM22 and α-SMA. Scale bars, 50 μm (white) and 10 μm (yellow). Arrows indicate double positive cells. (**B**) Representative flow cytometric analysis of tdTomato^{+} and α-SMA^{+} cells from aortic grafts (n=4).

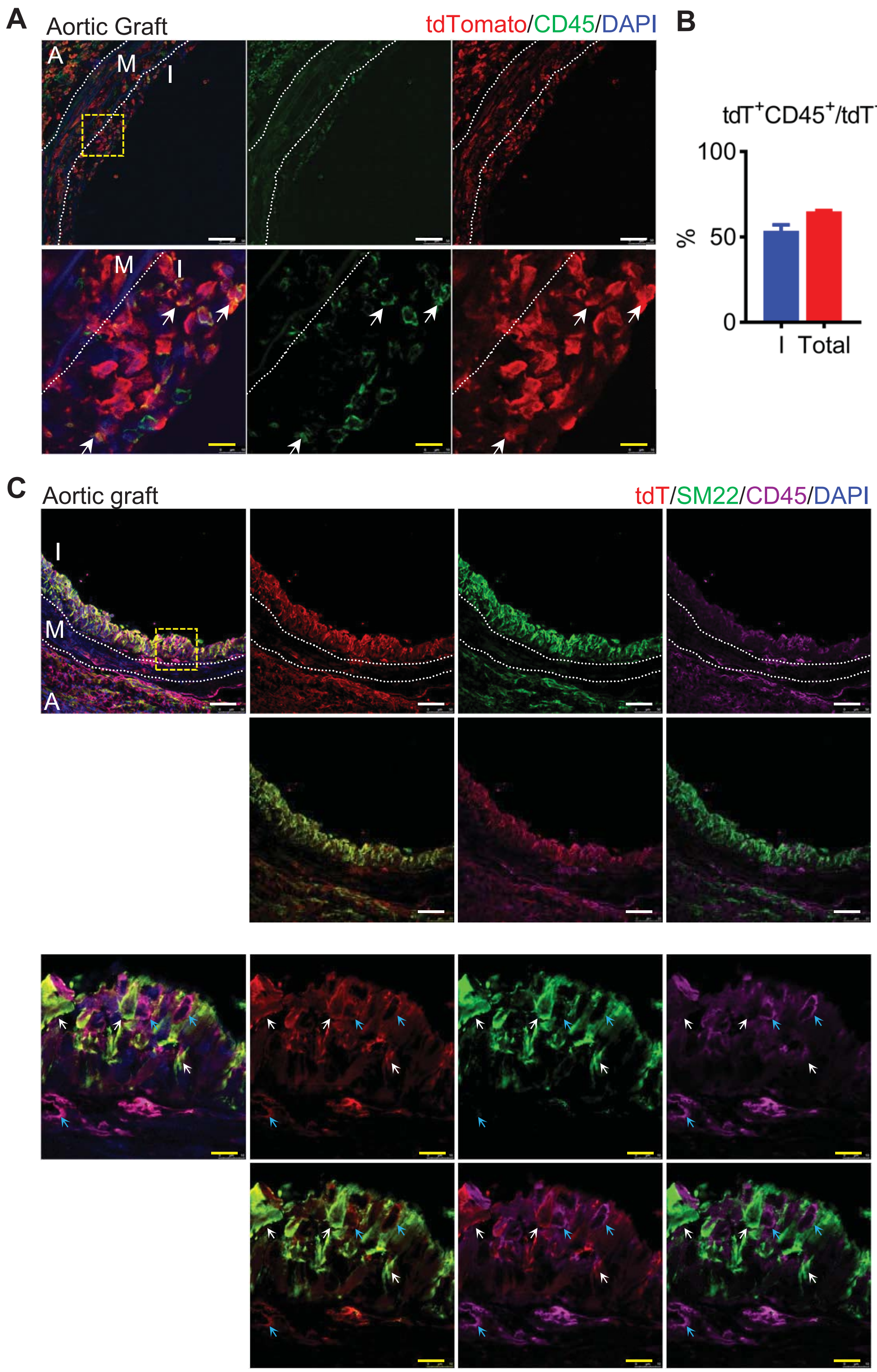

Online Figure IX. Related to Figure 2. tdTomato⁺CD45⁺ cells in aortic allograft. Aortic grafts were isolated from Kit-CreER; Rosa26-tdTomato mice described in Figure 2A. (**A**) Representative images showing aortic allograft stained with tdTomato and CD45. Scale bars, 50 µm (white) and 10 µm (yellow). Arrows indicate $tdTomato^{+}CD45^{+}$ cells. (**B**) Quantification of $tdTomato^{+}$ and $CD45^{+}$ cells in the neointima and the whole aortic graft. Data represent mean ± SEM, n=6. (C) Representative images showing staining of tdTomato, SM22 and CD45 in the aortic graft. White arrows indicate examples of $tdTomato^{+}$ $SM22^{+}$ cells, blue arrows indicate examples of $tdTomato^{+}$ $CD45^{+}$ cells. Scale bars, 50 µm (white) and 10 µm (yellow). A indicates adventitia; M, media; I, neointima.

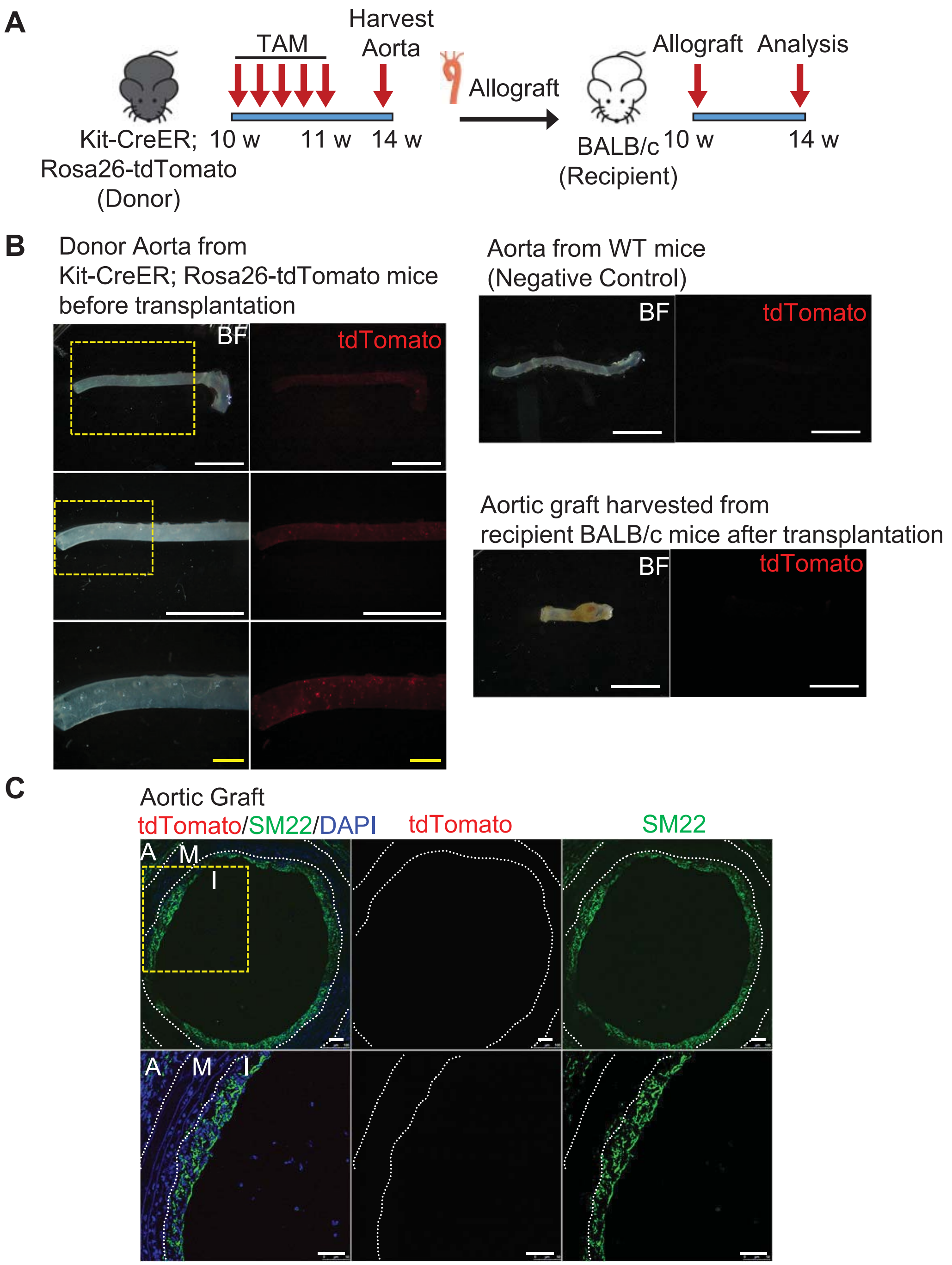

Online Figure X. c-Kit-derived neointimal SMCs are not from donor aorta. (**A**) Schematic showing procedure for allograft transplantation experiment. Kit-CreER; Rosa26-tdTomato mice were pulsed with tamoxifen and aortas were harvested 4 weeks later. Donor aortic segments were then transplanted into BALB/c mice and analysed 4 weeks later (n=6 mice per group). (**B**) Whole-mount brightfield and fluorescence images showing tdTomato labeling in donor aorta harvested from tamoxifen-treated Kit-CreER; Rosa26-tdTomato mice before transplantation, and aortic graft harvested from recipient BALB/c mice after transplantation. Aorta from wildtype mice served as negative control. Scale bars: 5 mm (white), 1 mm (yellow). (**C**) Representative images showing aortic grafts stained with tdTomato and SM22**.** Scale bars, 50 µm. BF indicates brightfield; A, adventitia; M, media; **I**, neointima.

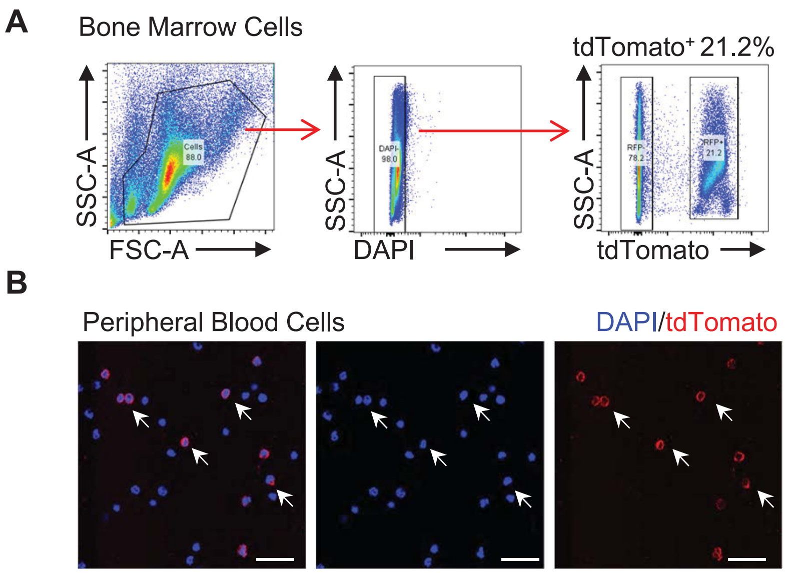

Online Figure XI. Related to Figure 3. Verification of tdTomato labeling in chimeric mice. (**A**) Representative flow cytometric analysis of bone marrow cells from chimeric mice (n=6 mice per group). (**B**) Representative immunostaining images of peripheral blood cells stained with tdTomato from chimeric mice (n=6 mice per group). Scale bars, 25 μm. Arrows indicate tdTomato^{+} cells.

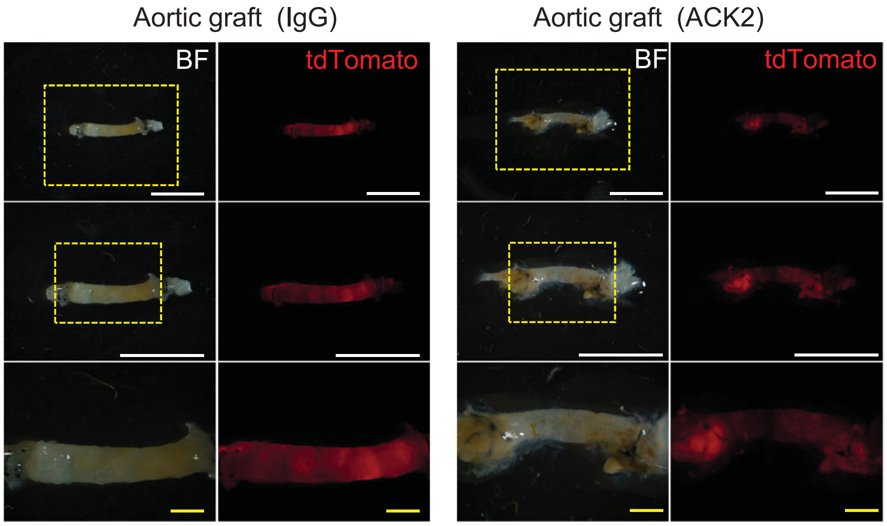

Online Figure XII. Related to Figure 4. Whole mount brightfield and fluorescence images showing tdTomato expression in aortic grafts with adjacent carotid arteries. Aortic grafts with adjacent carotid arteries were harvested from the mice described in Figure 4A (n=3). Scale bars: 5 mm (white), 1 mm (yellow).

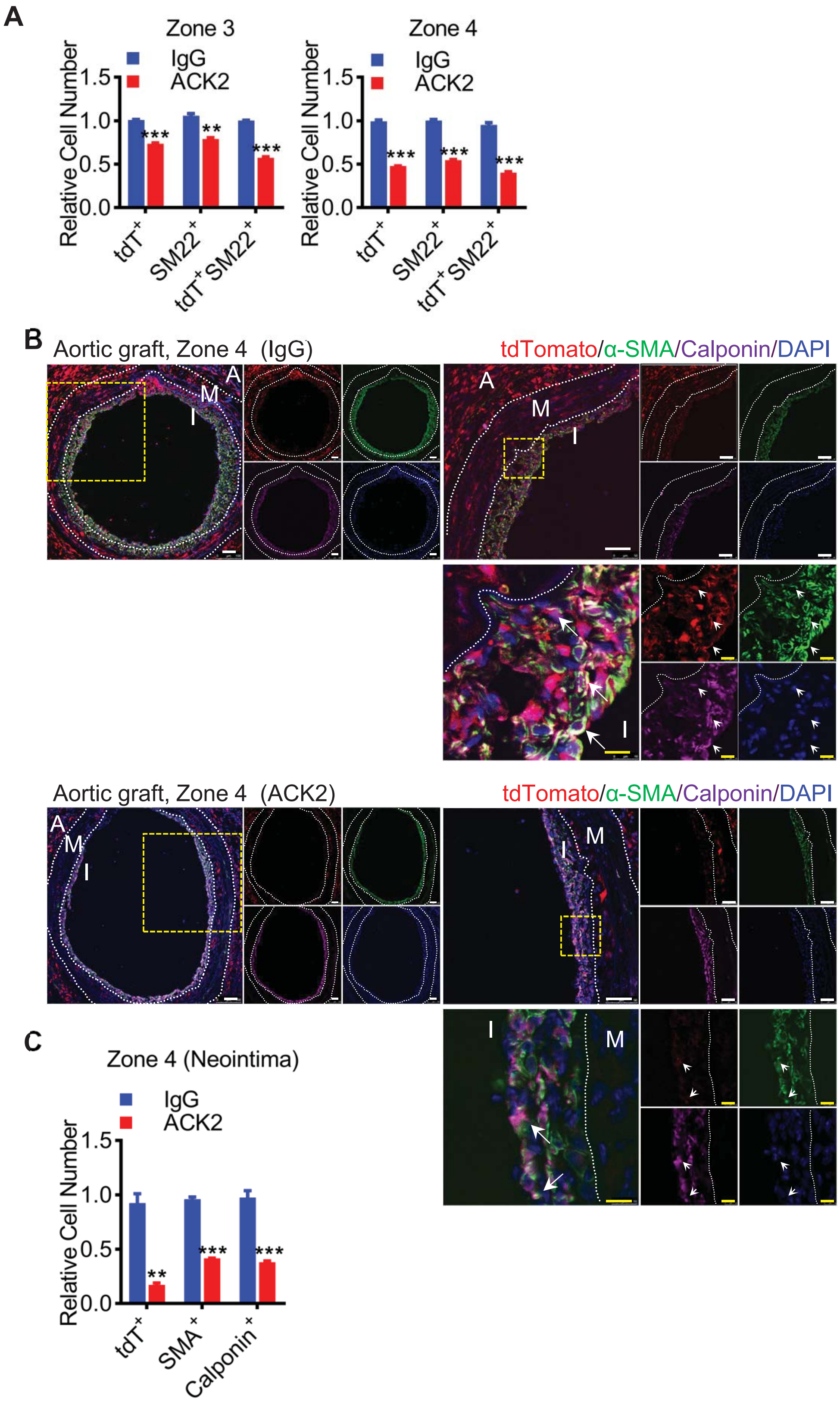

Online Figure XIII. Related to Figure 4. ACK2 reduces SMC accumulation in allograft-induced neointima formation. (**A**) Graph showing quantification of total tdTomato$^+$ cells and SM22$^+$ cells from aortic allografts in Figure 4B (n=3). (**B**) Representative images showing staining of tdTomato, α-SMA and Calponin in aortic grafts from IgG and ACK2-treated mice. Arrows indicate co-staining cells. Scale bars, 50 μm (white), 10 μm (yellow). (**C**) Graph showing quantification of tdTomato$^+$ cells, SMA$^+$ cells and Calponin$^+$ cells in neointima of allografts in **B** (n=3). Data represent mean ± SEM. **P<0.01, ***P<0.001, by unpaired two-tailed t test (**A** and **C**). A indicates adventitia; M, media; I, neointima; tdT, tdTomato.

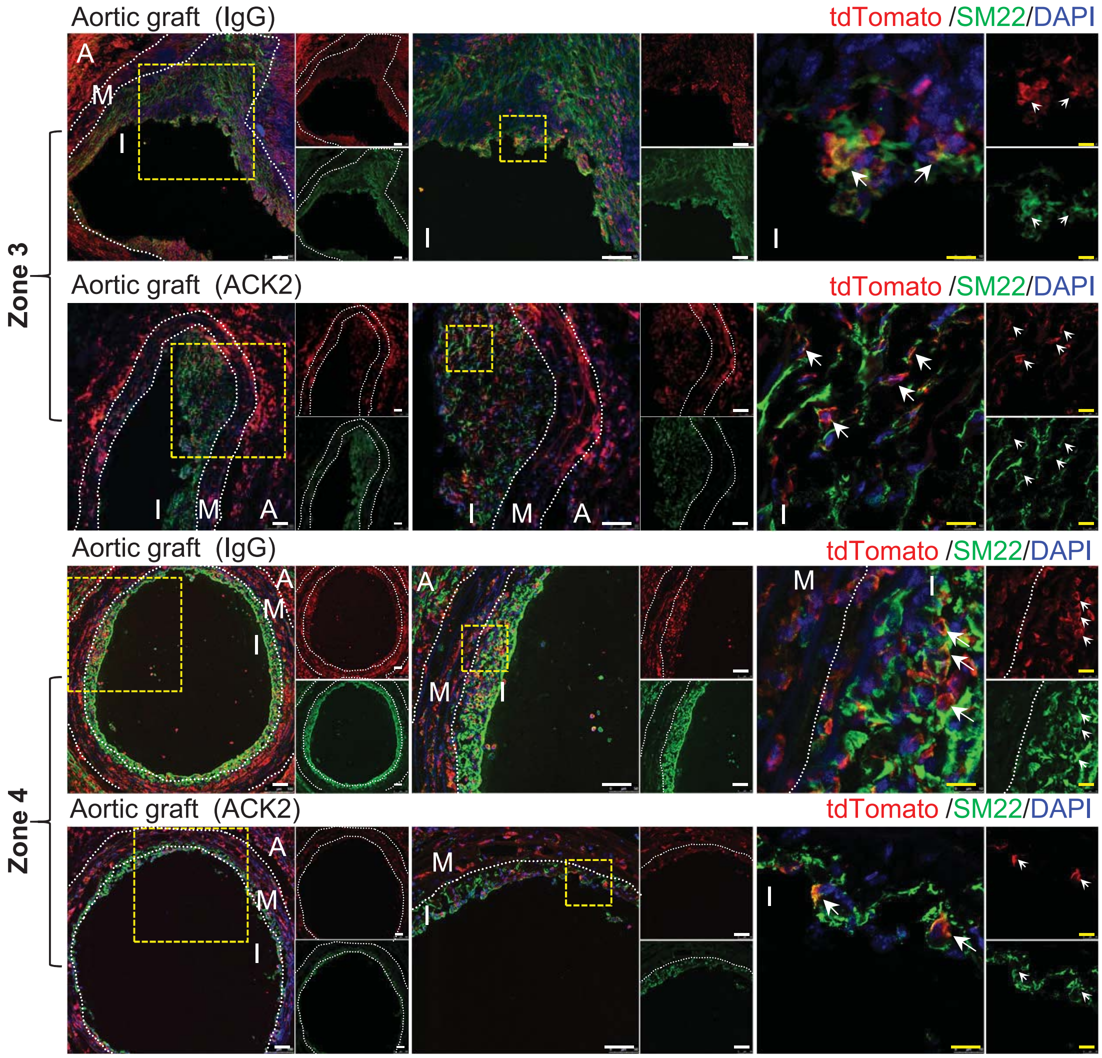

Online Figure XIV. Related to Figure 4. Blocking of c-Kit⁺ cells by ACK2 ameliorates transplant arteriosclerosis. Representative images showing staining of tdTomato and SM22 in Zone 3 and Zone 4 of the aortic graft from control IgG or ACK2 treated mice (with lower percentage of tdTomato-labelled neointimal SMCs compared to Figure 4). Arrows indicate examples of co-staining cells. Scale bars, 50 µm (white) and 10 µm (yellow). A indicates adventitia; M, media; I, neointima.

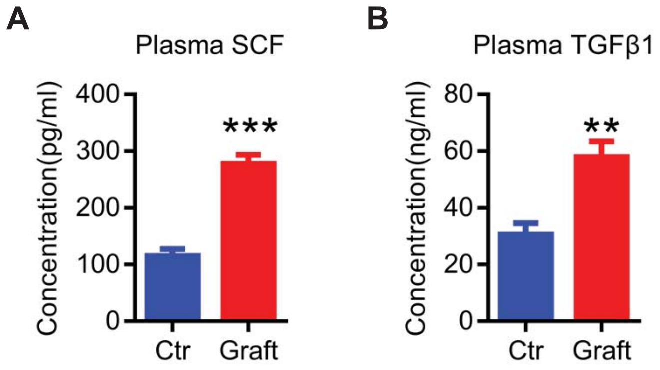

Online Figure XV. Quantification of SCF and TGFβ1 levels in blood plasma from control and allograft mice. The graphs indicated concentration of SCF (**A**) and TGFβ1 (**B**) in blood plasma of Kit-CreER; Rosa26-tdTomato mice described in Figure 1B, and allograft mice described in Figure 2A. Data shown are mean ± SEM, **P<0.01, ***P<0.001, unpaired two-tailed t test (**A, B**), n=6-7 mice per group. Ctr indicates control group.

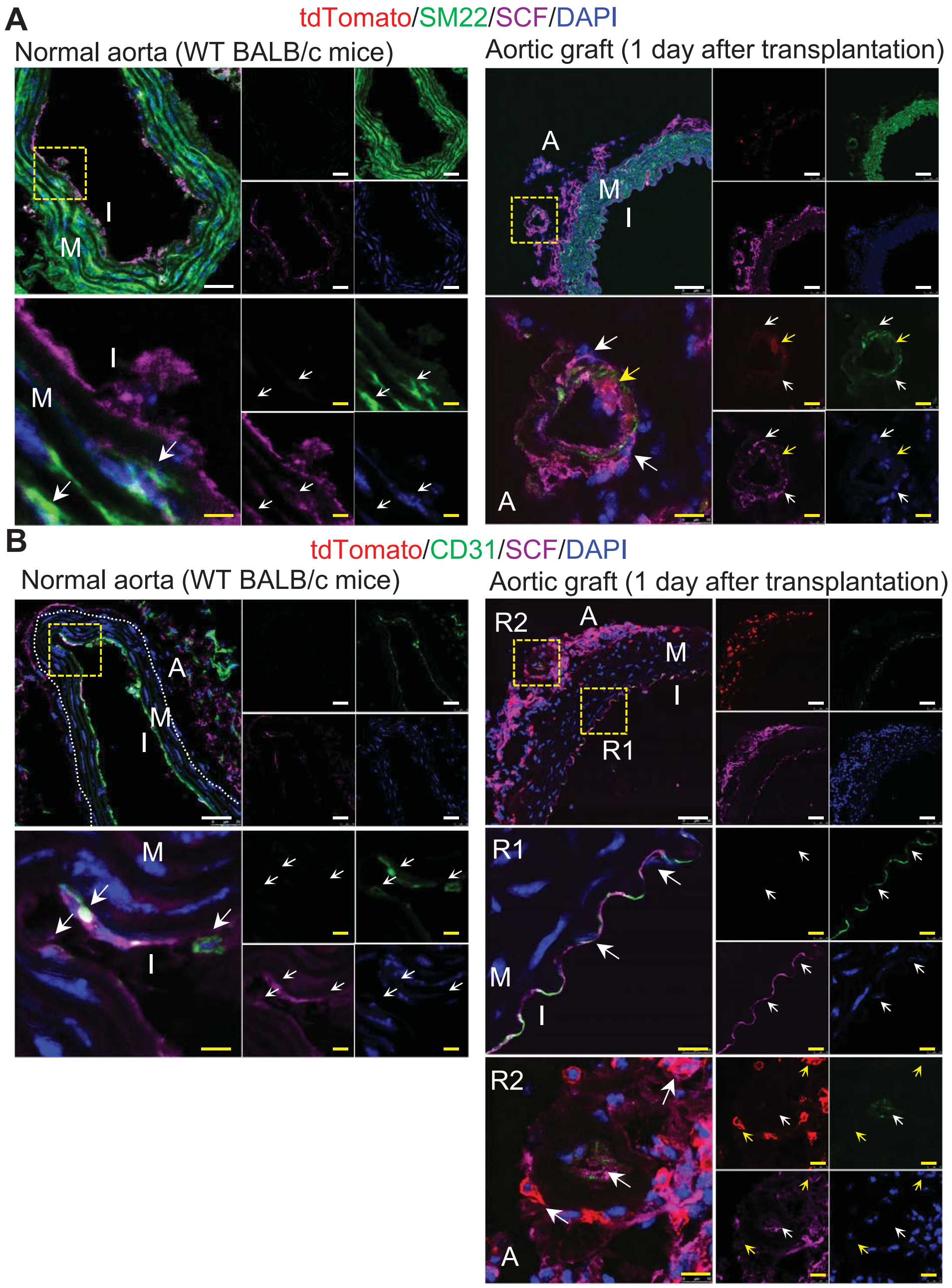

Online Figure XVI. Related to Figure 5A. The distribution of SCF in normal aorta, and aortic graft one day after transplantation. Normal aorta was harvested from wildtype BALB/c mice, while aortic graft (from donor BALB/c mice) was harvested from Kit-CreER; Rosa26-tdTomato mice one day after allograft surgery. (**A**) Representative images showing tdTomato, SM22 and SCF staining in normal aorta and aortic graft one day after transplantation (n=3 per group). (**B**) Representative images showing tdTomato, CD31 and SCF staining in normal aorta and aortic graft one day after transplantation (n=3 per group). White arrows indicate co-staining cells, yellow arrows indicate tdTomato^{+} cells. Scale bars, 50 μm (white), 10 μm (yellow). A indicates adventitia; M, media; I, intima or neointima; R1-2, region1-2.

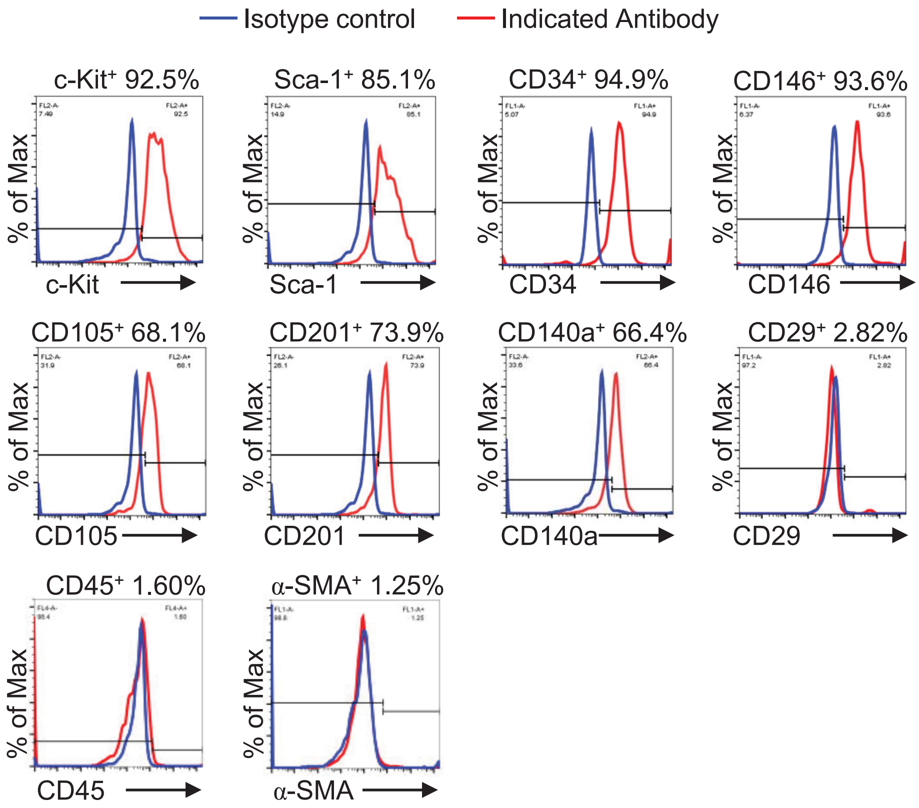

Online Figure XVII. Phenotyping of cultured c-Kit+ cells isolated from graft tissues. Cells were analysed by flow cytometry for their expression of surface markers including c-Kit, Sca-1, CD34, CD146, CD105, CD201, CD140a, CD29, CD45 and α-SMA. Corresponding IgG was used as isotype controls (n=3).

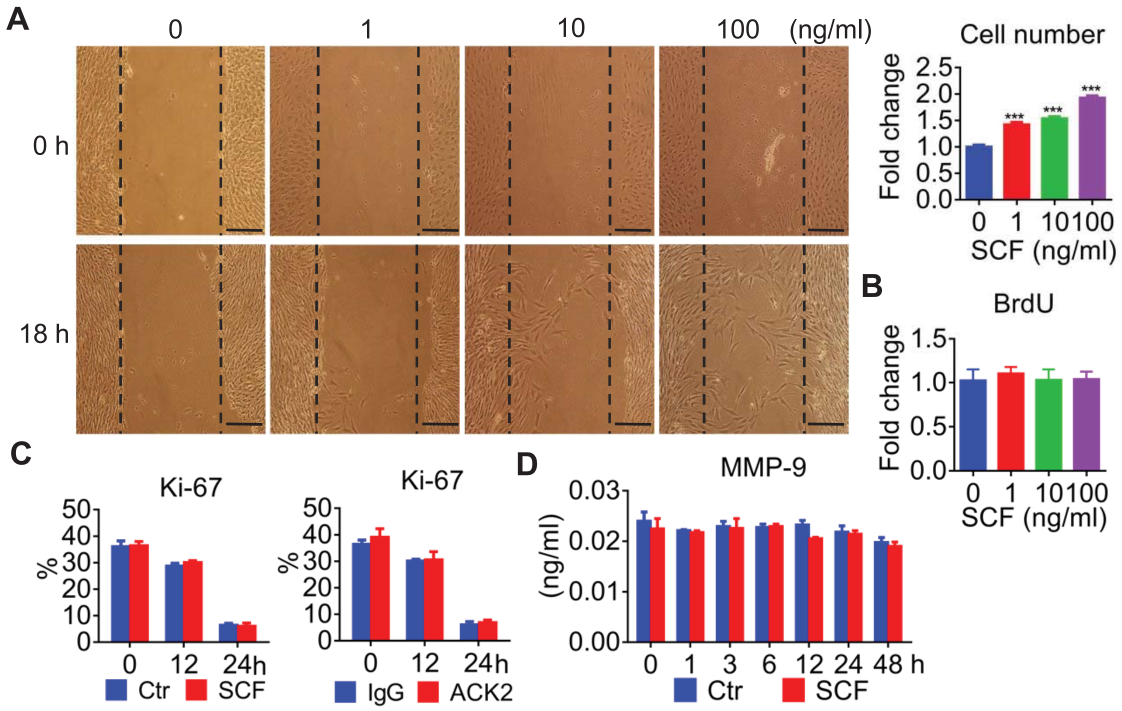

Online Figure XVIII. Cell migration, proliferation and MMP-9 secretion in c-Kit⁺ cells exposed to SCF. (**A**) Representative images showing migration of c-Kit⁺ cells in response to SCF using a scratch-wound assay. Scale bars, 100 µm. Graph are shown as fold change normalised to 0 h time point (n=5). (**B** and **C**) Cell proliferation was measured by BrdU assay (**B**) and Ki-67 staining (**C**) in SCF-treated c-Kit⁺ cells in the presence of ACK2 or IgG (n=3-4). (**D**) MMP-9 concentration in cell culture medium from SCF-treated c-Kit⁺ cells (n=3). All data shown are mean ± SEM. ****P*<0.001, by one-way ANOVA with Dunnett's test (**A** and **B**), and two-way ANOVA with Bonferroni's test (**C** and **D**). Ctr indicates control group.

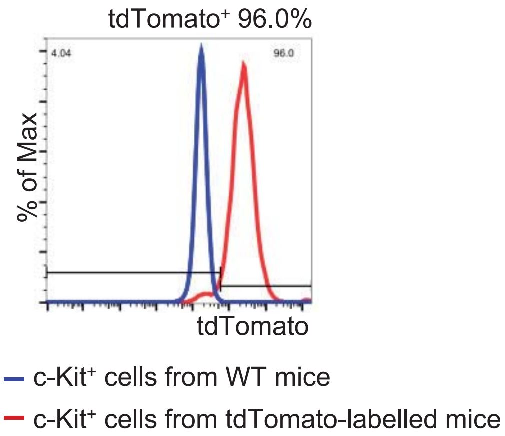

Online Figure XIX. tdTomato expression in c-Kit$^+$ cells isolated from graft tissues of Kit-CreER; Rosa26-tdTomato mice. Representative flow cytometric analysis showing tdTomato expression in c-Kit$^+$ cells isolated from wildtype and Kit-CreER; Rosa26-tdTomato mice (n=3).

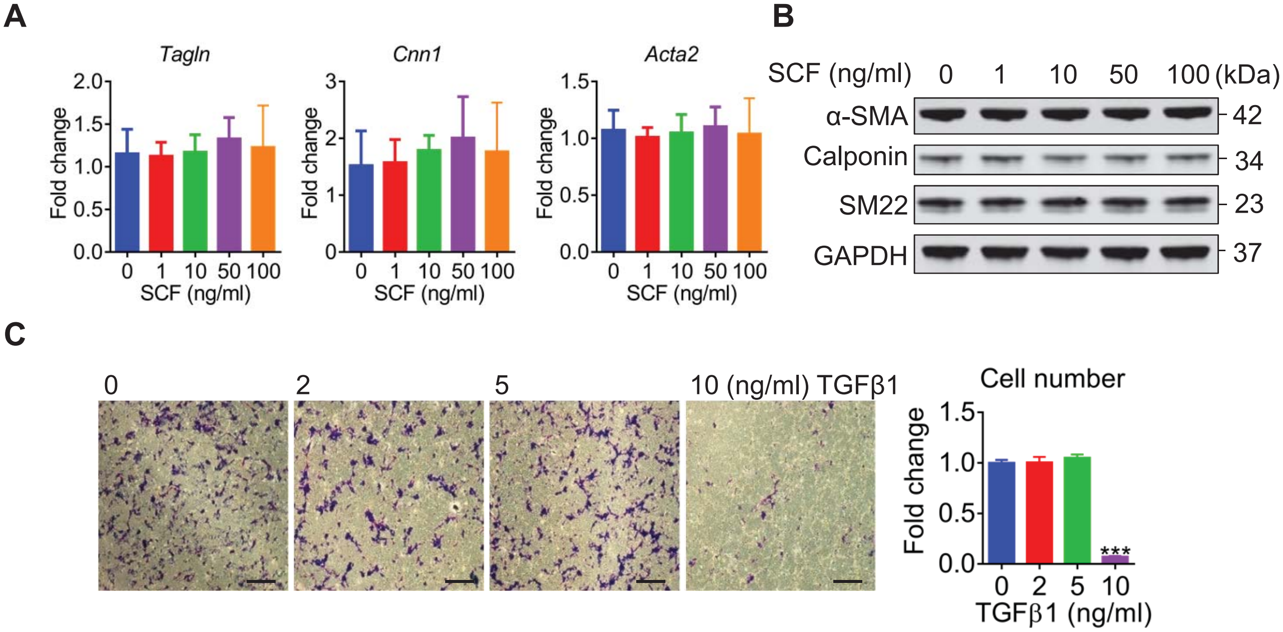

Online Figure XX. SCF does not induce expression of SMC markers, while TGFβ1 does not induce cell migration of c-Kit⁺ cell in vitro. (**A** and **B**) c-Kit⁺ cells were treated with indicated doses of SCF for 3 days. (**A**) qPCR analyses for mRNA expression of smooth muscle markers in cells treated with indicated doses of SCF (n=3 per group). (**B**) Representative western blot of smooth muscle markers in c-Kit⁺ cells treated with SCF (n=3). (**C**) Representative transwell migration images showing c-Kit⁺ cell migration in response to indicated doses of TGFβ1 (Scale bars, 100 μm). Graph was shown as fold change compared to control group (0 ng/ml TGFβ1), n=5 per group. Data represent mean ± SEM. ****P*<0.001, one-way ANOVA with Dunnett's test (**A** and **C**).

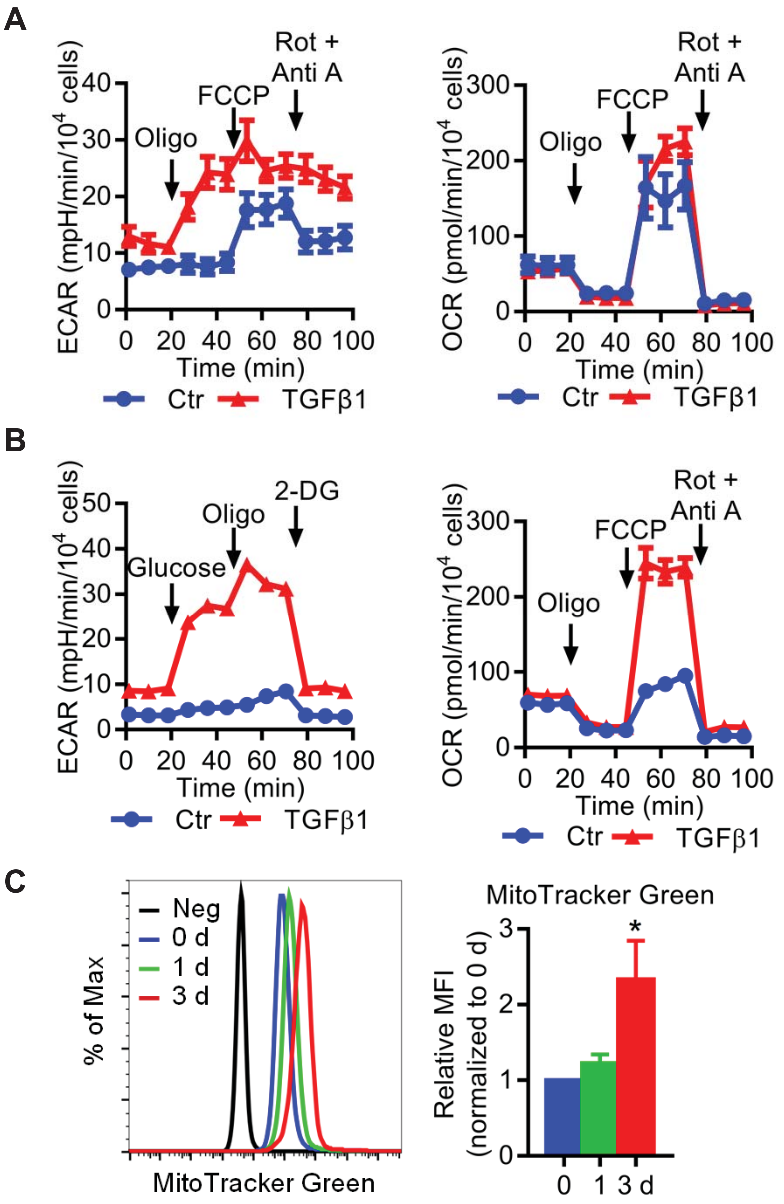

Online Figure XXI. Related to Figure 6. Metabolic reprogramming in c-Kit⁺ cells in response to TGFβ1. c-Kit⁺ cells were treated with TGFβ1 for 1 day (**A**), 3 days (**B**) or indicated times (**C**). (**A**-**B**) The ECAR over time was measured at basal level and after the injection of Oligo, FCCP, Rot and Anti A in **A**, or the injection of glucose, Oligo, and 2-DG in **B** (n=3-4 in **A**, n=6-7 in **B**). The OCR over time was measured at basal level and after the injection of Oligo, FCCP, Rot and Anti A (n=3-4 in **A** and **B**). (**C**) Representative histogram and quantification of relative MitoTracker Green MFI in c-Kit⁺ cells treated with TGFβ1 for indicated times by flow cytometry. Data shown are mean ± SEM. *P<0.05, one-way ANOVA with Dunnett's test, n=3 per group. Neg indicates negative control; Ctr, control; Oligo, oligomycin; Rot, rotenone; Anti A, antimycin A.

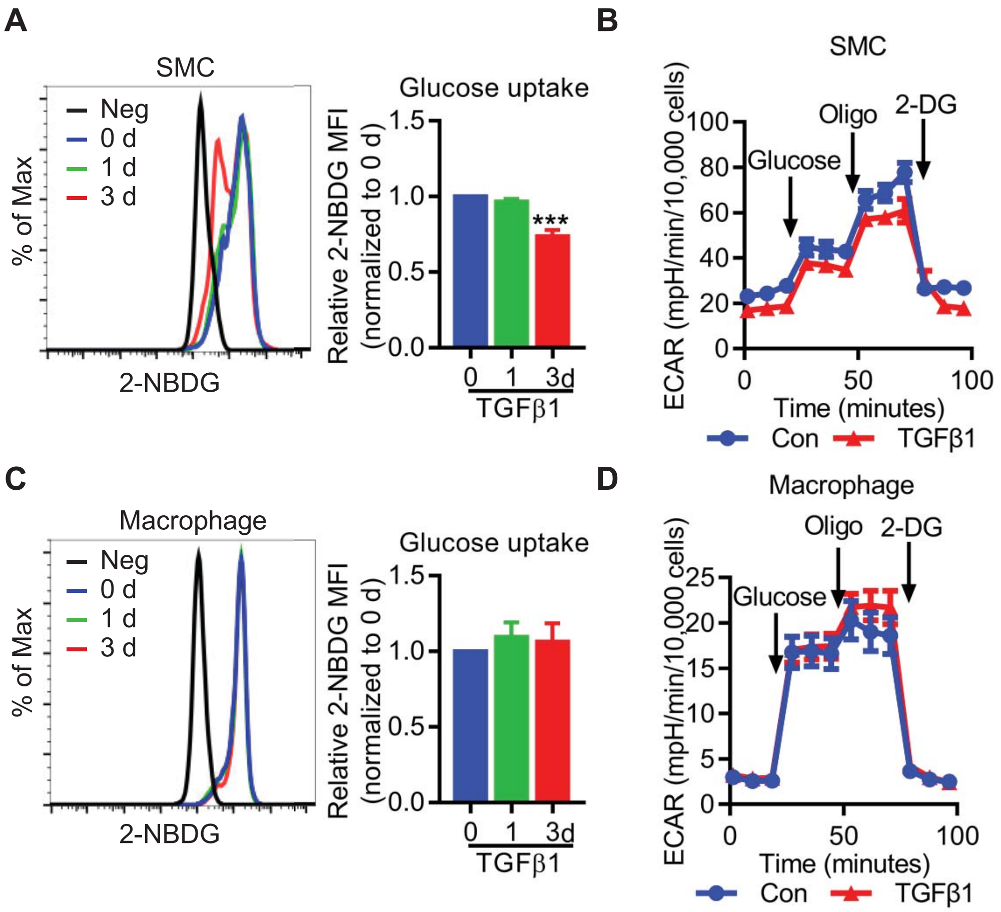

Online Figure XXII. The effect of TGFβ1 on SMC and Macrophage. Mouse aortic SMC and peritoneal macrophage were treated with TGFβ1 for indicated times (**A, C**) or 3 days (**B, D**). Representative histogram and quantification of 2-NBDG uptake in SMC (**A**) and macrophage (**C**) treated with TGFβ1 for indicated times by flow cytometry (n=3). The ECAR over time was measured at basal level and after the injection of glucose, Oligo, and 2-DG in SMC (**B**) and macrophage (**D**) (n=3). Data shown are mean ± SEM. ***P<0.001, one-way ANOVA with Dunnett's test, n=3 per group.

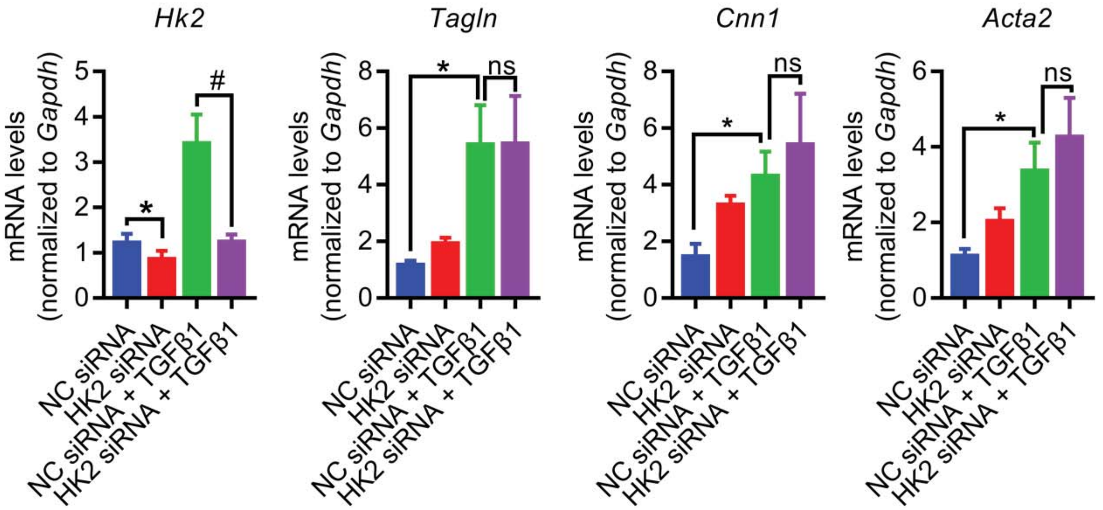

Online Figure XXIII. Knockdown of *Hk2* does not affect TGFβ1-induced c-Kit⁺ cell differentiation into SMC. qPCR analyses for mRNA expression of *Hk2* and smooth muscle markers in c-Kit⁺ cells transfected with NC or HK2 siRNA, treated with or without TGFβ1 for 48 h. Data shown are mean ± SEM. **P*<0.05, #*P*<0.05, ns indicates not significant, one-way ANOVA with Tukey's test, n=4 per group. NC siRNA indicates negative control siRNA.

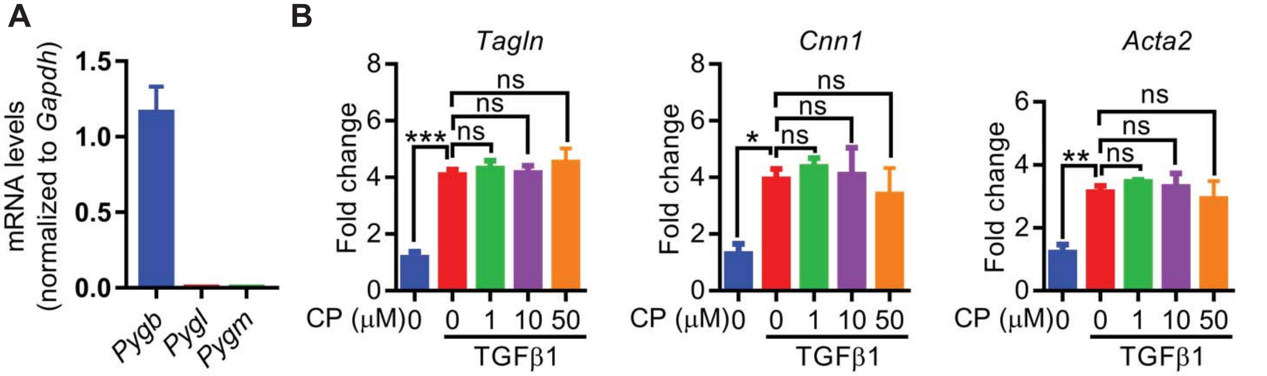

Online Figure XXIV. Glycogenolysis is not involved in TGFβ1-induced c-Kit⁺ cell differentiation into SMC. (**A**) qPCR analyses for mRNA expression of genes regulating glycogenolysis (*Pygb*, *Pygl* and *Pygm*) in cells (n=11 per group). (**B**) qPCR analyses for mRNA expression of representative smooth muscle markers in c-Kit⁺ cells treated with or without TGFβ1 (2 ng/ml) and indicated doses of CP-91149 for 48 h. All data shown are mean ± SEM. *P<0.05, **P<0.01, ***P<0.001, ns indicates not significant, one-way ANOVA with Tukey's test, n=4 per group. CP indicates CP-91149.

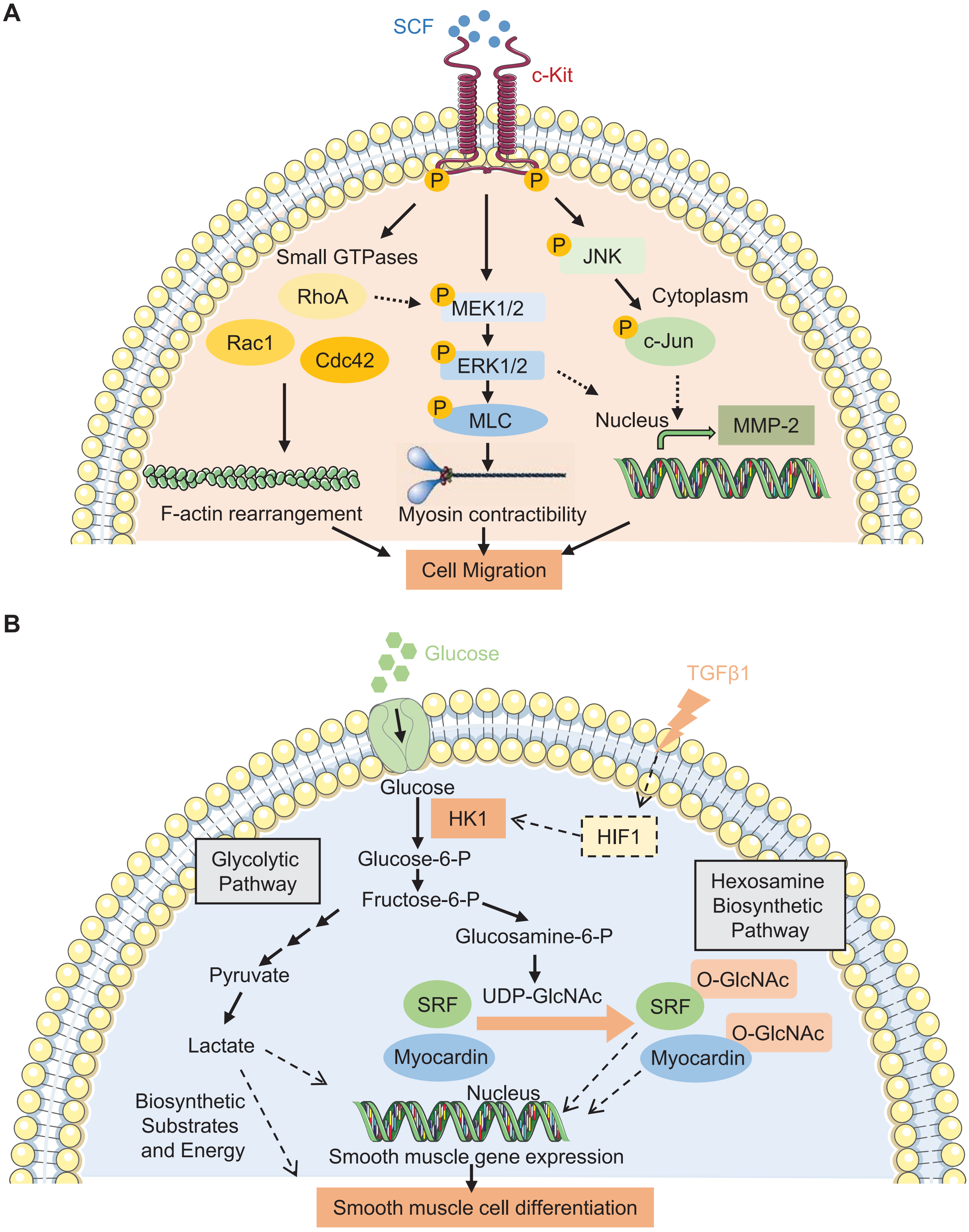

Online Figure XXV. Schematic diagram illustrating possible mechanisms regulating c-Kit⁺ cell migration and differentiation. (**A**) SCF stimulates c-Kit phosphorylation and induces activation of downstream small GTPases, MEK/ERK and JNK/c-Jun pathways. Activation of Rac1 and Cdc42 facilitates F-actin rearrangement to form cell protrusion. MLC can be phosphorylated through MEK/ERK signaling and increases myosin contractibility. Activation of the MEK/ERK and JNK/c-Jun pathways may further promote MMP-2 expression. These signaling pathways activated by the SCF/c-Kit axis concomitantly contribute to cell migration. (**B**) TGFβ1 increases glucose uptake and activates glucose metabolism in c-Kit^{+} cells. TGFβ1 may increase HK1 expression via HIF1. HK1-dependent glucose metabolism leads to activation of downstream glycolytic pathway and hexosamine biosynthetic pathway. Glycolytic pathway may provide biosynthetic substrates and energy for cell growth and differentiation. Hexosamine biosynthetic pathway provides UDP-N-acetylglucosamine (UDP-GlcNAc), a substrate for protein O-GlcNAcylation, to increase O-GlcNAcylation of SRF and myocardin, which may further regulate smooth muscle gene expression.
