# Supplementary figures and images for: Recipient c-Kit Lineage Cells Repopulate Smooth Muscle Cells of Transplant Arteriosclerosis in Mouse Models
Source: Circ Res. 2019 May 13;125(2):223–41. doi: 10.1161/CIRCRESAHA.119.314855 (PMC6615935; doi:10.1161/CIRCRESAHA.119.314855)

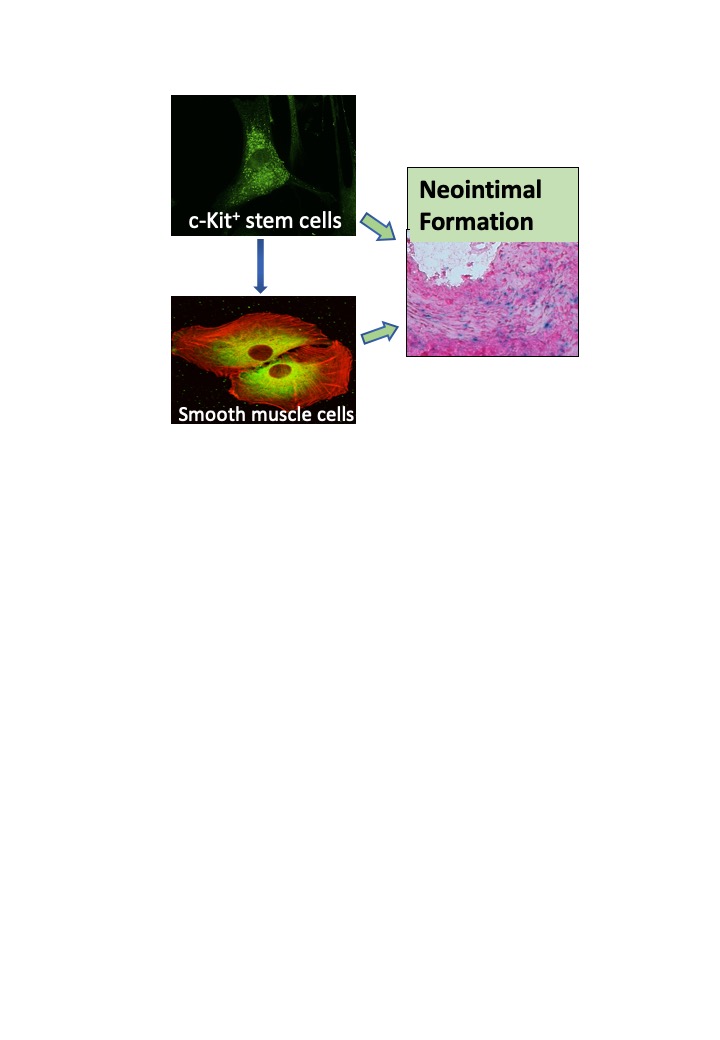

Supplement: Supplementary file 3 [file res-125-223-s003.jpg]
